# Structural Rheology in the Development and Study of Complex Polymer Materials

**DOI:** 10.3390/polym16172458

**Published:** 2024-08-29

**Authors:** Sergey O. Ilyin

**Affiliations:** A.V. Topchiev Institute of Petrochemical Synthesis, Russian Academy of Sciences, 29 Leninsky Prospect, 119991 Moscow, Russia; s.o.ilyin@gmail.com; Tel.: +7-(916)-8276852

**Keywords:** rheology, polymer solutions, specific interactions, polymer gels, gelation, phase separation, structure formation, polymer composites, chain architecture, polymer blends

## Abstract

The progress in polymer science and nanotechnology yields new colloidal and macromolecular objects and their combinations, which can be defined as complex polymer materials. The complexity may include a complicated composition and architecture of macromolecular chains, specific intermolecular interactions, an unusual phase behavior, and a structure of a multi-component polymer-containing material. Determination of a relation between the structure of a complex material, the structure and properties of its constituent elements, and the rheological properties of the material as a whole is the subject of structural rheology—a valuable tool for the development and study of novel materials. This work summarizes the author’s structural–rheological studies of complex polymer materials for determining the conditions and rheo-manifestations of their micro- and nanostructuring. The complicated chemical composition of macromolecular chains and its role in polymer structuring via block segregation and cooperative hydrogen bonds in melt and solutions is considered using tri- and multiblock styrene/isoprene and vinyl acetate/vinyl alcohol copolymers. Specific molecular interactions are analyzed in solutions of cellulose; its acetate butyrate; a gelatin/carrageenan combination; and different acrylonitrile, oxadiazole, and benzimidazole copolymers. A homogeneous structuring may result from a conformational transition, a mesophase formation, or a macromolecular association caused by a complex chain composition or specific inter- and supramolecular interactions, which, however, may be masked by macromolecular entanglements when determining a rheological behavior. A heterogeneous structure formation implies a microscopic phase separation upon non-solvent addition, temperature change, or intense shear up to a macroscopic decomposition. Specific polymer/particle interactions have been examined using polyethylene oxide solutions, polyisobutylene melts, and cellulose gels containing solid particles of different nature, demonstrating the competition of macromolecular entanglements, interparticle interactions, and adsorption polymer/particle bonds in governing the rheological properties. Complex chain architecture has been considered using long-chain branched polybutylene-adipate-terephthalate and polyethylene melts, cross-linked sodium hyaluronate hydrogels, asphaltene solutions, and linear/highly-branched polydimethylsiloxane blends, showing that branching raises the viscosity and elasticity and can result in limited miscibility with linear isomonomer chains. Finally, some examples of composite adhesives, membranes, and greases as structured polymeric functional materials have been presented with the demonstration of the relation between their rheological and performance properties.

## 1. Introduction

The past three decades have been marked by two events in polymer science: the active beginning of using nanoparticles to modify polymer materials [1,2,3] and the synthesis of new macromolecular objects with complex structures and nonlinear architectures, such as multiblock copolymers [4,5], dendrimers [6,7], star-shaped polymers [8,9], and other branched and highly branched macromolecules [10,11,12,13], which can also be utilized to enhance the properties of industrially produced linear polymers. However, the initial boom in nanotechnology for the polymer industry ended in partial disappointment due to the failure to achieve the theoretically substantiated results in improving mechanical strength and other characteristics of plastics [14,15]. High aggregation of nanoparticles and the absence of objective criteria for assessing the reasons and grades of their aggregation/disaggregation were raising issues, leading to scattered information about the success of applying nanomaterials, which also include nanofiltration membranes [16,17], improved greases [18,19,20], adhesives [21,22], and other functional multi-component systems besides polymer nanocomposites [23,24,25]. At the same time, macromolecular nano-objects for modifying the properties of plastics and creating new materials were almost not used at all due to the limitations in synthesis volume and relative costliness. Nevertheless, the potential of nanoparticles, multiblock copolymers, and highly branched macromolecules is enormous due to the theoretically attainable extremely high interfacial area in blends with usual polymers, possible self-assembly, and nanostructuring [26,27,28,29,30].

Objective criteria are necessary for evaluating the dispersibility and structure formation of nano-modifiers in polymer matrices, including modifiers of nonlinear macromolecular structure, whose phase state in blends with common linear polymers is poorly studied. Rheometry can serve as an efficient assessment method that is extremely sensitive to the structures and phase states of complex materials [31,32,33]. Rheological studies can provide exhaustive information about the suitable methods of mixing and processing of polymer-based materials, including details of the necessary mechanical impact conditions for disaggregating the modifier particles, since their current structure and dimensionality manifest themselves in the viscosity and viscoelasticity of composites [34,35,36]. Structural rheology involves establishing the relation between the material’s structure, the structure and properties of the material’s elements, and its rheological behavior as a whole [37,38], thereby naturally prompting its choice as a working tool for the creation and study of complex polymer materials, including novel nanomaterials with various applications and improved operational properties.

**Figure 1:**
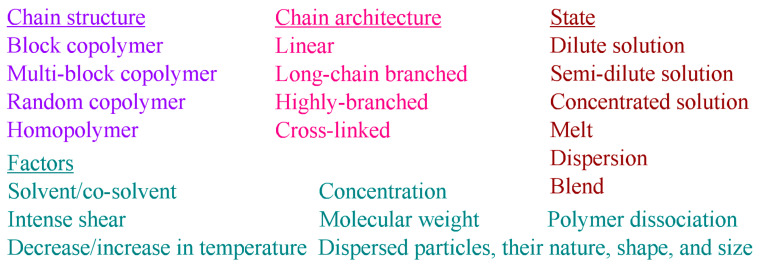
Objects of the study.

This generalized study aims to determine the fundamental correlations between the rheological properties and the structure of complex macromolecular systems for subsequent use in developing novel functional materials with improved performance, including polymer greases, membranes, composites, and adhesives. In turn, the aim requires detecting the conditions for forming micro- and nano-heterogeneous structures in complex polymer materials and identifying the manifestations of these structures via systematic structural rheology investigations. A set of objects and situations was considered (Figure 1), including possibilities of structure formation of polymers of different structures (block, multi-block, and random copolymers and homopolymers) with different chain architectures (linear, long-chain branched, highly branched, and cross-linked) in their dilute, semi-dilute, and concentrated solutions; melts; dispersions; and blends under the influence of different factors—solvent composition or addition of cosolvent, intensive mechanical shear, increase or decrease in temperature, change in concentration and molecular weight of the polymer, its dissociation, and addition of dispersed particles of different natures and shapes.

## 2. Rheological Approaches to the Study of Polymer Materials

A determination of the composition of a polymer material, its structure, and phase state based on its rheological properties in melts and solutions is the reverse task of rheology, created as a new field of physics for colloid chemists on the initiative of Eugene Bingham and Markus Reiner at the end of the 1920s [37,39,40]. Almost simultaneously, polymer science arose thanks to the first works by Hermann Staudinger, which included the study of the intrinsic viscosity of polymers [41,42]. Polymer science, like any other, develops towards complexity: new synthesis techniques lead to novel macromolecular objects and their combinations—complex polymer materials [43]. Complexity means nonlinearity, lack of subordination to known simple laws, interaction of simple components, and the appearance of new properties. Complexity may lie in the structure and architecture of the macromolecular chain [44,45], in specific intermolecular interactions [46,47,48], in the unusual phase behavior of macromolecules [49,50,51,52,53,54], and in the structure of a multi-component material, whose complex structure is typical for novel polymer lubricating greases, adhesives, membranes, and composites [16,17,18,19,20,21,22]. Their production is usually through the realization of a fluid state, where the task of rheology is to establish the method and conditions of processing a polymer material based on its rheological characteristics that must connect to its composition and structure by structural rheology. Starting with Bingham’s work on viscoplastic media [55], rheology primarily considers two fundamental orthogonal properties of a material—the abilities to shape recovery (elasticity) and irreversible deformation (flow and plasticity), most often by measuring viscosity and studying linear viscoelasticity.

### 2.1. Viscosity

Capillary and rotational viscometries are the primary two methods to measure viscosity [56,57]. In both cases, there is shear deformation of a sample, but the rotational method forcing it to flow between stationary and rotating surfaces has several influential advantages. It requires much lower sample volume, allows for flexible variation of deformation modes within a broader range (shear rate ($\dot{\gamma }$) can be in the range of 10^−5^–10^4^ s^−1^, and shear stress (*σ*) is within 10^−3^–10^4^ Pa), permits the study of viscoelasticity (viscometry becomes rheometry), provides greater accuracy of measurements, and makes it possible to study fluids with complex rheological behavior in a uniform shear field (using a cone/plate pair). The fundamental advantage of capillary viscometry is modeling the conditions for shaping polymer melts via extrusion and injection molding [58]. Similarly essential are tests of polymer melts and solutions by uniaxial stretching when it is necessary to predict their behavior under fiber- or film-shaping conditions [59,60,61]. Nevertheless, precision rotational rheometers have found widespread use due to their universality of application, relative simplicity of operation, and serial production by several world manufacturers (TA Instruments (New Castle, DE, USA), Thermo Scientific HAAKE (Karlsruhe, Germany), Anton Paar Physica (Graz, Austria), Netzsch Kinexus (Selb, Germany)), almost displacing other rheometric equipment in scientific and factory labs.

Rotational rheometry includes two main methods of material investigation. The first one is viscosity measurement during steady-state flow when a shear stress applies to the sample through a rotating surface and the resulting shear rate is measured, or vice versa [56,57]. Newton’s law allows for calculating the viscosity of a simple inelastic fluid: *η* = *σ*/$\dot{\gamma }$. At a transition to a non-Newtonian fluid, this equation will only be valid when using a measuring cone/plate or cone/cone unit, as exclusively a conical surface ensures uniform shear stress throughout the sample volume. A plate/plate pair, Couette coaxial cylinders, or a capillary require complex equations to calculate the apparent viscosity of non-Newtonian fluids correctly [57], but work well for ones that obey a linear Newton’s law.

**Figure 2:**
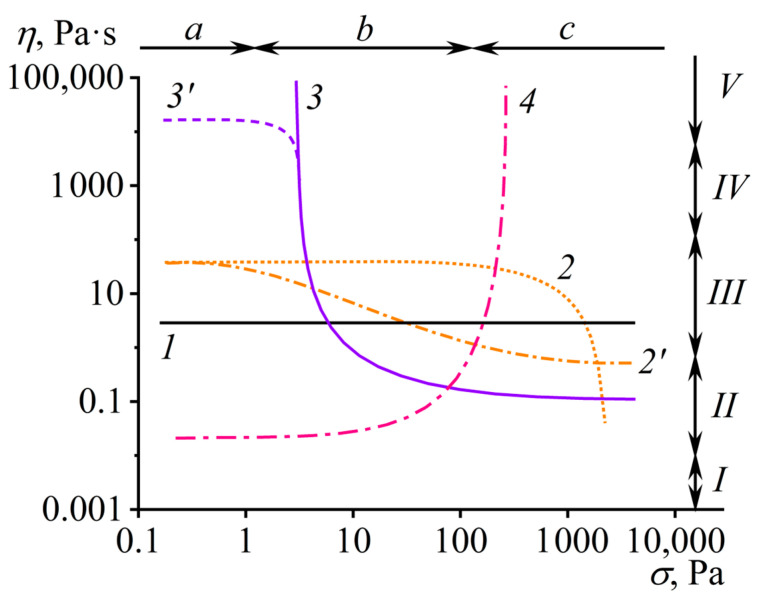
A generalized flow diagram of materials characterized by Newtonian behavior (*1*), pseudoplasticity (shear-thinning) typical for polymer melts/concentrated solutions (*2*) and colloid dispersions (*2*′), viscoplasticity of non-flowable strong gels (*3*) and prone-to-creep soft gels (*3*′), or dilatancy (shear-thickening) (*4*). The diagram’s right side shows the viscosity ranges inherent to volatile polymer solvents (*I*); easily flowing liquids such as base oils, plasticizers, and dilute polymer solutions (*II*); moderately flowing liquids, including oligomers and concentrated polymer solutions (*III*); poorly flowing liquids such as polymer melts (*IV*); and systems in a rubbery or glass-like state (*V*). The top side indicates viscosity drop zones due to the reduction in density of weak van der Waals and supramolecular interactions (*a*), stronger intermolecular hydrogen bonds and interparticle coagulation contacts (*b*), and interparticle phase contacts and macromolecular entanglements (*c*).

In the simplest case, the measured viscosity does not depend on the applied shear rate or shear stress, changing linearly following Newton’s law. Such Newtonian behavior is inherent to low-molecular-weight liquids (including multi-component ones) at temperatures significantly above their glass transition temperature, dilute colloid dispersions, non-concentrated macromolecule solutions, and melts of monodisperse polymers [62]. Non-Newtonian behavior manifests differently [63]: the apparent viscosity rises or declines with the shear rate and shear stress within their specific range. The apparent viscosity may also change in time at a constant applied shear rate or shear stress: increase (a material exhibits rheopexy) or decrease (thixotropy) [64,65]. Measurement of viscosity as a material property should occur in steady-state conditions, i.e., at high observation times when the phenomena of rheopexy and thixotropy have no effect. Figure 2 shows typical *η*(*σ*) curves when testing materials of different natures [57,63].

Two system types exhibit viscosity reduction with increasing shear rate or shear stress (shear-thinning behavior): pseudoplastic and viscoplastic (Bingham [55]) [63,66,67]. The apparent viscosity of pseudoplastic systems may decrease across the entire shear stress range (curve *2*′, e.g., due to the gradual escalating disaggregation of dispersed particles, their orientation, or elongation in the case of emulsions) or only at high shear stresses (curve *2*, typical for concentrated polymer solutions and polydisperse polymers in melts because of the orientation of deformed macromolecular chains and reduction in entanglement density). Viscoplastic systems have yield stress: a shear stress or range of shear stresses at which the sample’s apparent viscosity sharply drops due to the destruction of its spatial structure [68,69,70], while lower shear stresses do not allow the sample to flow (curve *3*) or may cause its creep, but with very high apparent viscosity (zero-shear, low-shear, or highest Newtonian viscosity, curve *3*′).

Dilatant systems exhibit a rise in the apparent viscosity with increasing shear rate (shear-thickening behavior, curve *4*), typically associated with intensified interactions of dispersed particles at high flow rates due to their friction, which may also cause jamming—a flow stoppage due to structure formation that causes yield stress behavior to arise [71,72,73]. In reality, basic types of rheological behavior may combine and manifest individually under different deformation conditions. A viscoplastic sample may flow with low apparent viscosity under the elevated shear stresses that are higher than its yield stress, but dilatantly become solid upon increasing the shear stress and then gain fluidity again at even higher shear stresses [66,74,75]. In turn, all technological operations and processing equipment limit the material’s minimum and maximum possible viscosity, but can operate within a specific range of shear stresses and shear rates. These aspects make the knowledge of viscosity behavior at different deformation conditions critically necessary for equipment setting and successful material processing.

### 2.2. Linear Viscoelasticity

The second base method for investigating rheological properties is deformation at small amplitude oscillatory shear (SAOS) for studying linear viscoelasticity [56,57]. One of the measuring surfaces of a rheometer tangentially oscillates according to a harmonic equation with a specific angular frequency *ω* and an amplitude of shear strain *γ*_0_ or shear stress *σ*_0_. The amplitude of this input impact is selected to be so small as not to change a material structure. The sample’s responsive reaction contains its amplitude (*σ*_0_ or *γ*_0_, oppositely to the input) and the phase angle *δ,* representing a delay between the shear stress and shear strain oscillations. As a result, the SAOS method allows for determining a complex modulus G* = *σ*_0_/*γ*_0_, its dependence on the angular frequency, and its two orthogonal components: the storage modulus *G*′ = G*cosδ and the loss modulus *G*″ = G*sin*δ*.

Magnitudes of the storage and loss moduli, their ratio (*G*″/*G*′ = tan*δ*), and their frequency dependences determine the linear viscoelasticity of a material. The storage modulus characterizes the amount of energy accumulated by the material during deformation, indicating its elastic properties. The loss modulus determines how much energy dissipates within the material under the same conditions—its capacity for irreversible (plastic or viscous) deformation. The higher both moduli are, the more the material resists deformation. In addition, the more the storage modulus exceeds the loss modulus, the more reversible the material’s deformation under applied load and the fuller the material’s ability to recover its shape after removing the load. Both moduli may depend on the frequency of impact on the material, and studying the frequency dependencies of the moduli allows for an assessment of how a given material will behave under different impact frequencies or observation times *t* (since *t* = 1/*ω*).

**Figure 3:**
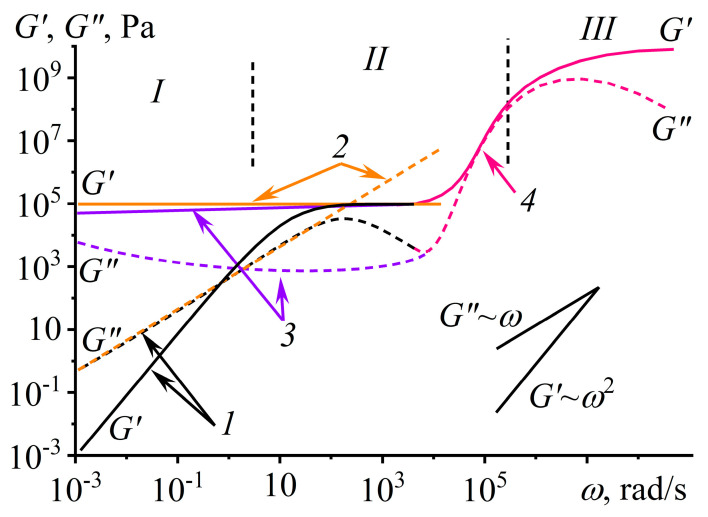
Frequency dependencies of storage modulus (*G*′, solid lines) and loss modulus (*G*″, dashed lines) of materials showing the behavior of Maxwell viscoelastic liquid (*1*); Kelvin–Voigt elastoviscous solid (*2*); solid-like gel (*3*); or a monodisperse polymer having three relaxation states (*4*): viscous (zone *I*, curves are the same as shown for *1*), rubbery (*II*), and glassy (*III*). Thus, polymer systems have one more relaxation state due to macromolecular entanglements. The inset shows the moduli’s slopes according to the Maxwell model in the terminal (low-frequency) zone. Potentially, any system is liquid-like at low frequencies of external actions (long observation times) and conversely becomes solid-like with an increase in the frequency, as reflected by the Deborah number—the ratio of the relaxation time to the time of the applied action or its observation *De* = *τ/t* [76,77].

For an inelastic liquid, *G*′ = 0 and *G*″ = *ηω* (*δ* = 90°), whereas *G*′ = *G*_0_ and *G*″ = 0 (*δ* = 0°) for a solid, where *η* and *G*_0_ are the Newtonian viscosity and the shear modulus, respectively. Polymer materials simultaneously exhibit both behavior types, and two basic models describe their linear viscoelasticity: the Maxwell model and the Kelvin–Voigt model. The Maxwell model represents a viscoelastic liquid behaving like a rubber or a glass at high frequencies with $G^{\prime }(\omega )=\frac{\tau^{2}\omega^{2}}{\tau^{2}\omega^{2}+1}G_{0}$ and $G^{\prime \prime }(\omega )=\frac{\tau \omega }{\tau^{2}\omega^{2}+1}G_{0}$ (Figure 3, curves *1*), where *τ* is the relaxation time (equal to *η/G*_0_) and *G*_0_ is the shear modulus in the rubber state or the glassy state. At low frequencies, *G*′~*ω*^2^ and *G*″~*ω*. The Kelvin–Voigt model represents an elastoviscous, rubbery-like solid capable of creeping with *G*′ = *G*_0_ and *G*″ = *ηω* (curves *2*) [56,57].

Polymer and colloid gels [78,79], being viscoplastic, do not conform to these models and behave similarly to a defective or amorphous (glassy) solid with *G*′ *≈ G*_0_ and *G*″ << *G*′ (curves *3*). Meanwhile, solutions and melts of polymers with narrow molecular weight distributions demonstrate low-frequency Maxwell behavior (viscous flow and then rubbery-like high-elasticity), which passes to glass-transition and glassy-state regions as the frequency increases, thus combining two parallel-connected Maxwell models (Burgers model [80], curves *4*). A description of more complex systems requires a combination of basic models, at least the spectral extension of Maxwell relaxation times with increasing polymer dispersity (*Đ* = *M*_w_/*M*_n_) [57].

The study of linear viscoelasticity allows for the determination of the behavior of polymer materials under low loads, providing information about their undamaged structure and network density. Simple networks can be of macromolecular entanglements, chemical cross-links, supramolecular non-covalent bonds, or dispersed particles’ coagulation contacts, and their density is the ratio *G*_0_/*RT*, where *R* and *T* are the universal gas constant and absolute temperature, respectively [81]. Moreover, the same rheological approaches allow for assessing the structural features of complex networks such as interpenetrating polymer networks [82,83]; composite gel networks [84,85]; emulsion-filled gel networks [86]; and novel covalent adaptable networks from dissociative reagents, associative vitrimers, or compounds able to post-cure irreversible chemical metamorphosis for creating different smart, self-healing, shape-morphing, and recyclable materials [87,88]. In addition, the steady-state viscosity of homogeneous polymer systems coincides with their complex viscosity ($\left|\eta^{*}\right|=\frac{\sqrt{{G^{\prime }}^{2}+{G^{\prime \prime }}^{2}}}{\omega }$) at numerically equal shear rates and angular frequencies. This well-known Cox–Merz rule [89] allows viscosity to be estimated under conditions not achievable for steady-state flow, e.g., because of shear-induced phase separation or Weissenberg effect appearance.

### 2.3. Nonlinear Viscoelasticity

**Figure 4:**
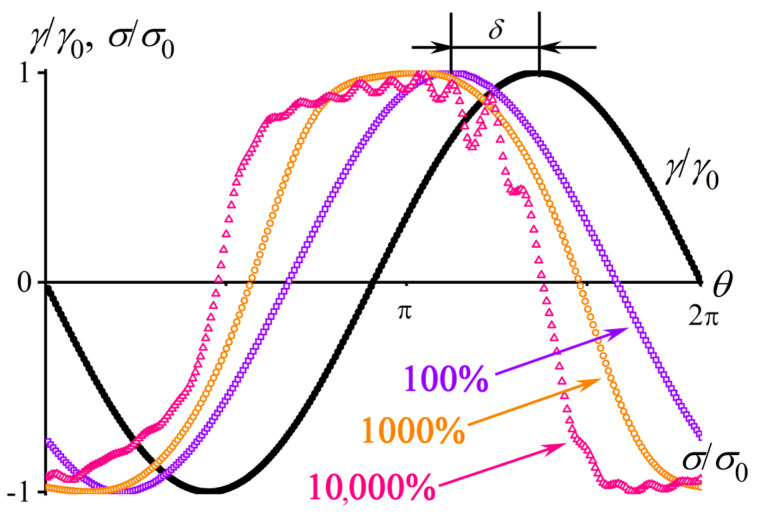
Normalized responses of polyethylene melt (*σ*/*σ*_0_) to a linear input action, the periodic deformation (*γ*), according to a harmonic law with different strain amplitudes (*γ*_0_) indicated near the curves (adapted from [91]).

However, it is necessary sometimes to know materials’ behavior under large deformations, which change their structure, e.g., because of stretching and orientation of macromolecular chains or destruction of a percolation network from filler particles. The study of nonlinear viscoelasticity applies large-amplitude oscillatory shear (LAOS) [90], which deforms a sample at a fixed angular frequency with increasing amplitude of shear strain or shear stress. The problem arises when a deformation exceeds a critical value, and the Hooke law is no longer valid (*σ*_0_/*γ*_0_ ≠ *const*): the linear input action (e.g., shear strain *γ*(*t*) = *γ*_0_sin(*ωt*)) no longer produces a material’s linear response (*σ*(*t*) ≠ *σ*_0_sin(*ωt + δ*)). This deviation from the pure sinusoidal response becomes more pronounced as the input action’s amplitude increases (Figure 4).

**Figure 5:**
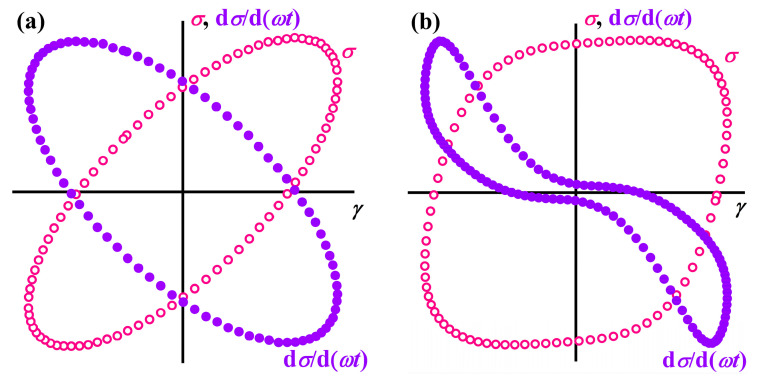
Lissajous figures with linear ((**a**), *γ*_0_ = 100%) and nonlinear ((**b**), *γ*_0_ = 1000%) responses of polyethylene melt (adapted from [91]).

The challenge lies in the correct calculation of the storage and loss moduli. The common practice involves the transformation of the nonlinear response into a Fourier series and calculating the moduli based on the amplitude and phase angle of the first harmonic [90]. At the same time, the neglect of higher harmonics is reasonable only for roughly estimating the nonlinear behavior of a material because they have substantial shares in its response (approximately 10% or 50% in total for a polymer melt when the strain amplitude is 1000% or 100,000%, respectively [92]). The incorporation of higher harmonics can be via Chebyshev polynomials of the first kind, which allows for calculating nonlinear storage and loss moduli at both minimum and large strains [93], but introduces noise from inaccuracy in determining the 5th and higher harmonics and does not take even harmonics into account [92]. A variant of the nonlinear analysis uses the Lissajous–Bowditch figure—the dependences of instantaneous shear stress on instantaneous shear strain [94,95]. In the general case of linear viscoelastic response, the figure in these coordinates is an ellipse (Figure 5a), described by the equation ${\left(\frac{\sigma }{\sigma_{0}}\right)}^{2}+{\left(\frac{\gamma }{\gamma_{0}}\right)}^{2}={(\sin\delta )}^{2}+2(\frac{\sigma }{\sigma_{0}})(\frac{\gamma }{\gamma_{0}})\cos\delta$. For a viscous liquid, the ellipse transforms into a circle while becoming a straight line for an elastic material [96]. The area of the ellipse (*A* = π*σ*_0_*γ*_0_sin*δ*) represents the part of the work completed during the material deformation cycle and irreversibly lost as heat. Its calculation based on experimental data allows the loss modulus $G\prime \prime =\frac{\sigma_{0}}{\gamma_{0}}\sin\delta =\frac{A}{\pi \gamma_{0}^{2}}$ [93] to be found. The evaluation of the storage moduli at minimum and large deformations utilizes the derivative of shear stress at zero shear strain *G*′_M_ = d*σ*/d*γ* (*γ* = 0) and Hooke’s law at the maximum strain *G*′_L_ = *σ*/*γ* (*γ* = *γ*_0_), respectively [93,97]. However, large strains (higher than 1000%) bring noise into the material response and, hence, the contours of the Lissajous figure [91], making the calculations irreproducible. In addition, *G*_L_ is an averaged modulus and does not truly reflect the viscoelasticity at the point of amplitude strain.

Numerical differentiation of the material response by the product *ωt* (d*σ*/d(*ωt*) = *σ*_0_cos(*ωt + δ*)) solved the problem of correctly finding the storage modulus at large strains [91,98]. In the Lissajous coordinates, the relationship of d*σ*/d(*ωt*) versus strain at small *γ*_0_ also turns out to be an ellipse whose area (*E* = π*σ*_0_*γ*_0_cos*δ*) determines the elastic energy accumulating (and releasing) during the periodic shear cycle: $G\prime =\frac{\sigma_{0}}{\gamma_{0}}\cos\delta =\frac{E}{\pi \gamma_{0}^{2}}$. The integrations of the traditional and differentially transformed Lissajous figures yield the nonlinear large-strain loss and storage moduli (*G*_L_), respectively. The calculations of minimum-strain moduli (*G*_M_) are also possible using the integral method via changes in the areas of Lissajous figures when the strain amplitude increases (*γ_n_* − *γ_n_*_−1_): *G*_M_ = 2*G*_L_ − *G*_R_, where $G_{R}=\frac{{G_{L,n}\times \gamma }_{n}^{2}-G_{L,n-1}{\times \gamma }_{n-1}^{2}}{\gamma_{n}^{2}-\gamma_{n-1}^{2}}$ is a new measure of nonlinear viscoelasticity—the instantaneous (or differential) storage or loss modulus characterizing viscoelasticity at the point of amplitude shear strain *γ* = *γ*_0_ [91,92,98].

**Figure 6:**
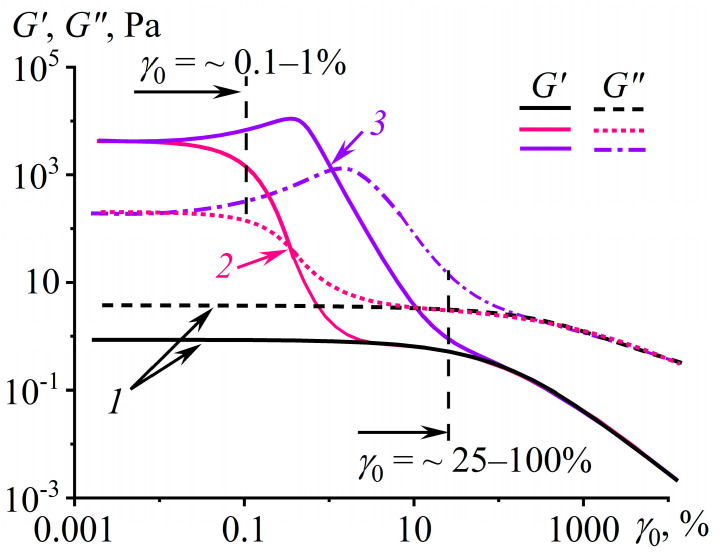
Amplitude dependencies of storage modulus (*G*′, solid lines) and loss modulus (*G*″, dashed lines) for Maxwell’s (*1*), viscoplastic (*2*), and dilatant (*3*) media. The vertical dashed lines represent the limit of the linear viscoelasticity region for different systems (*γ*_0_ where *G*′(*γ*_0_) ≠ *const*): smaller for structured colloids and longer for homogeneous polymeric and glass-forming liquids.

At large deformations, the dependencies of *σ* and d*σ*/d(*ωt*) lose their ellipse form (Figure 5b), which, nevertheless, does not hinder their numerical integration and finding the nonlinear storage and loss moduli [91,92,98]. In the case of simple glass-forming or macromolecular-entangled liquids, the nonlinearity in rheological behavior starts at a strain amplitude higher than 25–100%, manifesting as a decrease in both moduli (Figure 6, curves *1*). For the case of viscoplastic materials, the linear region is significantly shorter, and a decrease in the moduli occurs at *γ*_0_ = 0.1–1% (curves *2*). In turn, the loss modulus or both moduli of dilatant systems pass through a local maximum and then decrease (curves *3*). In summary, the use of the LAOS method expands the arsenal of rheological techniques for analyzing polymer materials, allowing for their rheo-classification [90], observation their shear-induced structure evolution [99,100], direct and accurate determination of the nonlinear storage and loss moduli, and, subsequently, the fitting parameters of rheological models [101]. Additionally, this method facilitates achieving high shear rates, which are not attainable in classic steady-state flow conditions due to the manifestation of the Weissenberg effect [99].

## 3. Complex Chain Structure and Its Manifestation in the Polymer Structuring

### 3.1. Microphase Separation of Block Copolymer Melts

**Figure 7:**
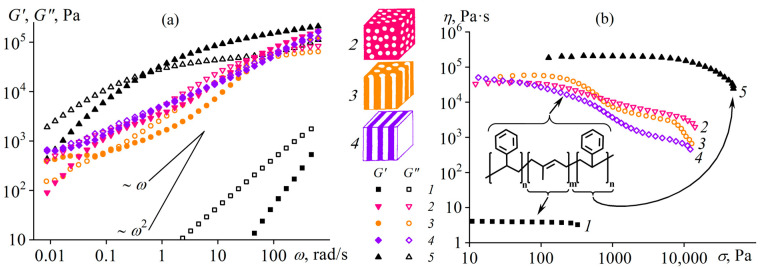
Storage and loss moduli versus angular frequency (**a**) and viscosity versus shear stress (**b**) at 170 °C: polyisoprene (*1*; 65 kDa), polystyrene (*5*; 184 kDa), and SIS containing 13 (*2*; 144 kDa, 12 nm), 18 (*3*; 140 kDa, 10 nm), or 44 wt% of styrene units (*4*; 82 kDa, 5 nm). The values in parentheses represent the number-average molecular weight (*M*_n_) and diameter or thickness of polystyrene domains (according to [103]). Schematic SIS microstructures are in the middle.

Structure formation in polymer melts can occur in the case of a block copolymer macromolecular structure [102]. For instance, lamellar, cylindrical, or spherical nanoscale domains form in a melt of a styrene–isoprene–styrene triblock copolymer (SIS), depending on its block ratio and temperature [103]. This microphase separation reflects itself in the rheological properties: the melt’s behavior does not correspond to the Maxwell model at low and moderate frequencies (Figure 7a). Spherical polystyrene domains in the SIS medium lead to a less pronounced frequency dependence of the moduli at moderate frequencies with liquid-like behavior of the melt (*G*″ > *G*′). In this case, *G*′~*ω*^2^ and *G*″~*ω* at *ω* → 0, like for polyisoprene and polystyrene homopolymer melts. The transition from spherical to cylindrical microdomains causes a solid-like behavior at moderate observation times (around 1/*ω ≈* 100 s), since *G*″ < *G*′ and *G*′ *≈ const* at low frequencies. A layer-structured melt has storage and loss moduli close in values at a wide frequency range with their tendency to become constants at low *ω*.

Extended lamellar or cylindrical spatial structures reflect themselves in the frequency independence of the storage modulus at low frequencies. Theoretically, *G*′~*ω*^2^ and *G*″~*ω* when angular frequencies are very low, as the polystyrene microphase represents a viscous liquid and transits to the terminal zone. The absence of yield stress (Figure 7b) confirms this relaxation transition of the structured melts behaving like Maxwell fluids at shear rates (and angular frequencies) less than 10^−5^–10^−3^ s^−1^. However, these low frequencies are beyond practical application due to lengthy testing times accompanied by polymer thermo-oxidation. Meanwhile, the microphase separation causes non-Newtonian behavior in block copolymer melts at lower shear rates and shear stresses than for homopolymers because of the disruption of the microdomain structure under these conditions. The initial reduction in viscosity due to the homogenization of the melt structure takes turns by the usual viscosity decrease resulting from the orientation and disentanglement of the macromolecular chains under more intense shear conditions.

### 3.2. Microphase Separation of a Multiblock Copolymer Melt

Microphase separation in block copolymers and the accompanying changes in their rheological properties are strongly evident. However, rheometry allows for detecting microphase separation in multiblock copolymers, e.g., vinyl acetate and vinyl alcohol [104,105]. The multiblock copolymer results from partial alkaline hydrolysis of polyvinyl acetate, while the random copolymer, as a comparison sample, results from acetylation of polyvinyl alcohol. In the case of an equimolar ratio of vinyl acetate and vinyl alcohol groups, the average block length in the multiblock copolymer is five monomer units in contrast to the two units typical for the random copolymer.

**Figure 8:**
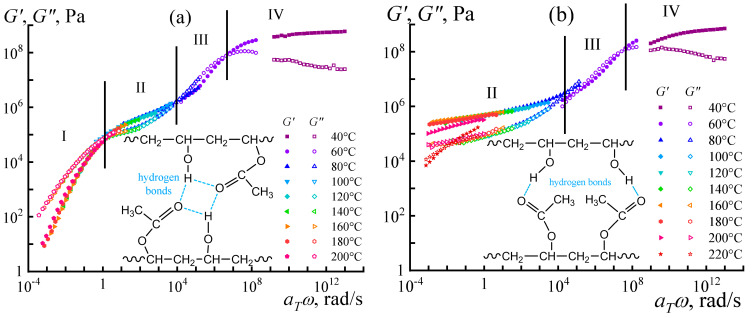
Frequency dependencies of storage and loss moduli measured at different temperatures and normalized to 120 °C for random (**a**) and multiblock (**b**) vinyl acetate/vinyl alcohol equimolar copolymers having polymerization degrees of 450. Viscoelastic regions represent terminal zone (I), rubbery plateau (II), glass transition (III), and glassy state (IV). The time–temperature superposition principle works well since the curves overlap (**a**), and vice versa (**b**). Insets show schematically intermolecular hydrogen bonds—simultaneously intra- and inter-chain (**a**) and predominantly inter-chain cooperative (**b**) (adapted from [104,105]).

The random copolymer in melt shows trivial rheological properties (Figure 8a). It follows the time–temperature superposition principle: rheological curves obtained at different temperatures merge to a single dependence that consists of viscous-flow, rubbery-state, glass-transition, and glassy-state regions [104]. The situation changes for the multiblock copolymer (Figure 8b). Firstly, the principle of time–temperature superposition fails, indicating the presence of at least two relaxation times that depend differently on temperature. One relaxation time is for the vinyl acetate blocks, while the other represents the vinyl alcohol blocks. Secondly, the multiblock copolymer does not have a terminal zone, at least at temperatures that do not cause noticeable thermo-oxidation. These particularities imply both microphase separation and crystallization of the vinyl alcohol blocks, likely due to strong cooperative interchain hydrogen bonds [106,107] resulting from sequentially arranged alcohol groups (inset in Figure 8b). Moreover, specific zipper-like interchain complexes may form, where two sequences of vinyl-alcohol units linked by parallel hydrogen bonds play the role of a zipper, while acetate units limiting them act as a zip holder [108]. In contrast, interchain hydrogen bonds of the random copolymer are non-cooperative and weak because of the formation of intrachain hydrogen bonds between neighboring alcohol and carbonyl groups along the chains (inset in Figure 8a) [105,109].

### 3.3. Macromolecular Association in a Multiblock Copolymer Solution

**Figure 9:**
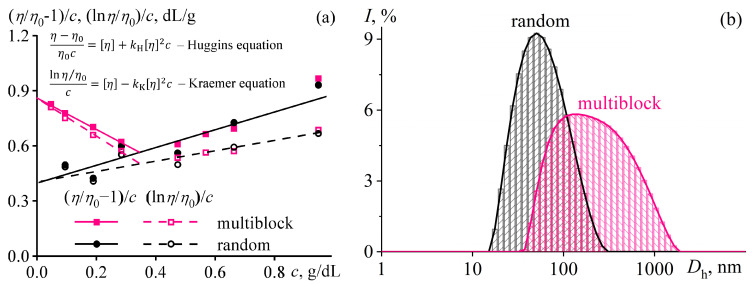
Concentration dependencies of the reduced viscosity of vinyl acetate/vinyl alcohol equimolar copolymers in DMF at 20 °C in the coordinates of the Huggins and Kraemer equations (**a**) and the size distribution of copolymer macromolecules and their associates by light scattering intensity in the solutions with copolymer concentrations of 0.28 g/dL (**b**) (adapted from [105]). The reduced viscosity for the random copolymer decreases with dilution (**a**), as for a usual polymer in its solution, whereas it goes through a minimum for the multiblock copolymer and then increases because of the growing size of the macromolecular associates, which are less likely to break down through mutual hydrodynamic action when their concentration becomes lower. The macromolecular association of the multiblock copolymer appears evident from the much larger sizes of its diffusing formations (**b**) consisting of dozens of individual chains.

Macromolecules of block structures tend to associate in solutions, leading to increased viscosity, non-Newtonian behavior, and gel formation [110]. However, the multiblock structure also manifests itself in copolymer solutions besides melts, both dilute and concentrated, e.g., in *N*,*N*-dimethylformamide (DMF) [105]. After accounting for the solvent viscosity (*η*_0_) and copolymer concentration (*c*), dilute solutions of the multiblock copolymer demonstrate an anomaly: dilution increases the reduced viscosity in the coordinates of the Huggins [111] and Kraemer [112] equations (Figure 9a). Such unusual behavior is absent for random copolymer solutions. The concentration dependencies of the reduced viscosity allow for assessing the macromolecular sizes by extrapolating the viscosity to a zero polymer concentration and determining the intrinsic viscosity [*η*] proportional to the cube of the mean hydrodynamic diameter of macromolecules *D*_h_ according to the Fox–Flory equation [113,114]. The intrinsic viscosity of the multiblock copolymer is twice as high, indicating that its macromolecules are in an associated form even in dilute solutions. Dynamic light scattering confirms this conclusion (Figure 9b). The average hydrodynamic diameter of diffusing objects for the random copolymer is 50 nm, which is possible for individual macromolecular coils in a good solvent. For the multiblock copolymer, the sizes of the objects are hundreds of nanometers, characteristic not of macromolecules, but their associates consisting of a dozen or more individual chains.

**Figure 10:**
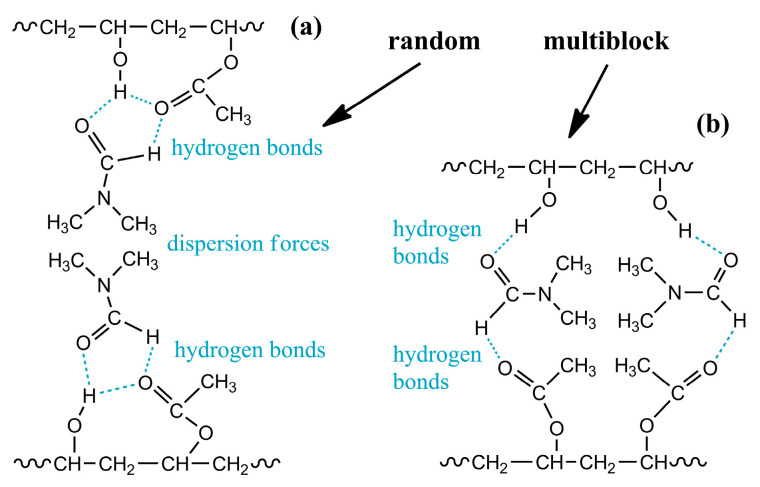
Interchain interactions of random (**a**) and multiblock (**b**) vinyl acetate/vinyl alcohol copolymers in *N*,*N*-dimethylformamide (adapted from [105]).

When copolymers dissolve, DMF molecules solvate them with the formation of hydrogen bonds. In the case of the random copolymer, the hydrogen bonds with the solvent are weak due to the alternating types of co-monomers along the chain and the formation of intramolecular hydrogen bonds of vinyl acetate groups with neighboring vinyl alcohol groups (Figure 10a). As a result, the solvent molecules form notional shells around the polymer chains, where polar groups face the chains and weakly polar methyl groups face outward. The surrounded chains then mutually interact only through weak dispersion interactions, and no chain associations occur [105]. In the case of the multiblock sample, hydrogen bonds virtually do not arise between neighboring monomers of the same type but form preferably with the solvent molecules (Figure 10b). For vinyl alcohol groups, these bonds supposedly are stronger than for vinyl acetate ones, but neighboring chains nevertheless can bind to each other through DMF molecules, leading to the association of multiblock macromolecules and the structuring of their solutions.

**Figure 11:**
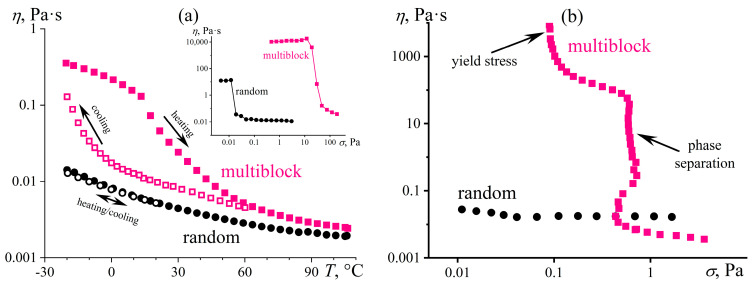
Temperature dependencies of viscosity for 5% solutions of equimolar vinyl acetate/vinyl alcohol copolymers in DMF at 100 s^−1^ (**a**) (the inset shows dependencies of viscosity on shear stress for these solutions at −20 °C) and shear stress dependencies of viscosity for 10% solutions of the same copolymers in DMF at 20 °C (**b**) (adapted from [105]). The viscosity of the random copolymer in its semi-dilute solution increases smoothly upon cooling, like for a usual polymer (**a**). In contrast, the viscosity rises in a jump-like manner for the multiblock copolymer because of the transition of its solution to the gel state, i.e., the arising of a yield stress of substantial value compared to the negligibly low yield stress of the weakly structured solution of the random copolymer (*σ*_Y_ of 19 Pa vs. of 0.012 Pa at −20 °C, see the inset). The transition to concentrated solutions (**b**) makes multiblock macromolecules metastable—non-flowable at low shear stresses and phase-separable at high ones, unlike the random copolymer having a nearly constant viscosity in the solution.

The structuring is evident in semi-dilute solutions characterized by an overlap of macromolecular coils [105]. The overall strength of interchain contacts for multiblock copolymers depends on the weakest bond, i.e., the weaker hydrogen bonds of vinyl acetate groups (Figure 10b). As a result, the total strength of the supramolecular network is insignificant. Nevertheless, the supramolecular binding of multiblock macromolecules through DMF molecules and hydrogen bonds leads to their higher viscosity in the semi-dilute solution in contrast to the random copolymer solution of the same concentration (Figure 11a). Furthermore, cooling strengthens hydrogen bonds and thus causes reversible gelation of the semi-dilute solution of the multiblock copolymer. There is a significant increase in viscosity with the arising of yield stress behavior (the inset to Figure 11a), unlike the solution of the random copolymer, which does not form a strong gel but only weakly structuralizes.

In concentrated solutions having chain entanglements, the cooperativity of hydrogen bonds and macromolecular association are also prominent (Figure 11b). The solution of the random copolymer behaves practically as a Newtonian liquid, whereas the viscosity curve of the multiblock copolymer solution exhibits two regions of constant shear stresses (indicated by arrows in Figure 11b). The first region corresponds to the yield stress—concentrated solutions of the multiblock copolymer do not flow at low shear stresses. The second region represents the macroscopic phase separation of the solution, initiated by intense shear. Intense shear causes the disentanglement and stretching of macromolecular chains, their mutual approach, and crystallization—the phase separation leads to a white precipitate. The dissolution of the multiblock copolymer occurs only at elevated temperatures (≈90 °C) that weaken interchain hydrogen bonds, and its concentrated solutions are in a metastable state [105].

## 4. Specific Interactions in Polymer Solutions and Their Structuring

The multiblock structure and, as a result, strong intermolecular hydrogen bonds lead to the structuring of both melts and solutions of vinyl alcohol copolymers. Meanwhile, structuring can also occur in the case of a random copolymer due to specific interactions of macromolecular chains with each other or with solvent molecules. However, specific macromolecular interactions in a polymer melt with subsequent structure formation are possible only for a complex chain structure, leading to mesophases of different types whose structure and rheology were previously studied in detail [115,116,117,118,119]. In other words, specific interactions of random-structure copolymers with macroisotropic structure formation are only possible in their solutions.

### 4.1. Supramolecular Structuring of a Random Copolymer’s Dilute Solution

**Figure 12:**
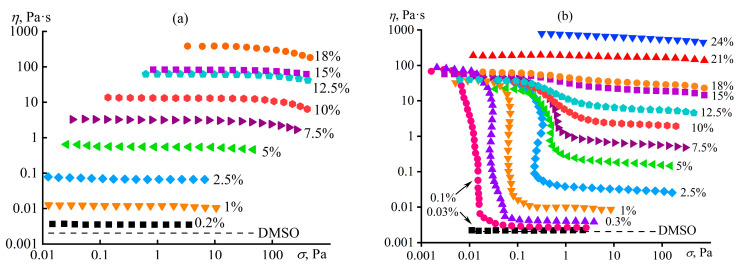

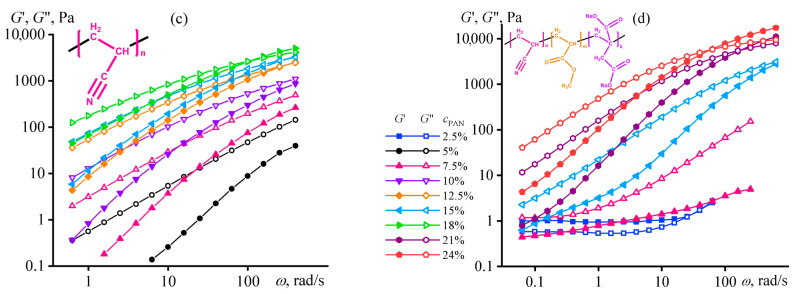
Shear stress dependencies of viscosity (**a**,**b**) and frequency dependencies of storage and loss moduli (**c**,**d**) for solutions of acrylonitrile homo- (**a**,**c**) and terpolymer (**b**,**d**) in DMSO at 20 °C. The polymer mass fraction is indicated at the curves or in the legend (adapted from [120,121]). The homopolymer in its solution exhibits non-Newtonian behavior only at high shear stresses and high concentrations (**a**), like an ordinary polymer, also showing the usual Maxwell viscoelasticity (**c**). In contrast, the terpolymer in solution changes behavior from the standard pseudoplasticity of a concentrated polymer solution to anomalous viscoplasticity typical for gels when its content declines (**b**), just as its viscoelasticity transforms from a Maxwellian liquid to a gel-like state upon dilution (**d**).

An example of supramolecular structuring is solutions of polyacrylonitrile (as a reference sample) and random terpolymer of acrylonitrile, methyl acrylate, and sodium itaconate (93.0/5.7/1.3 wt%) in dimethyl sulfoxide (DMSO), given their comparable molecular masses: *M*_n_ = 48 and 45 kDa, respectively (dispersity *Đ* = *M*_w_/*M*_n_ = 4.0 and 2.1) [99,120,121]. The homopolymer forms typical polymer solutions (Figure 12a), which exhibit weak non-Newtonian behavior only at high shear rates and high polymer concentrations (*c*_PAN_ > 5 wt%) because of the macromolecular disentanglement and decrease in the entanglement density. At the same time, terpolymer solutions demonstrate viscoplastic behavior even at *c*_PAN_ ≥ 0.1 wt%, and the higher the polymer concentration, the less pronounced the non-Newtonian flow (Figure 12b). A similar anomaly appears when studying linear viscoelasticity. The behavior of the homopolymer in DMSO is trivial: *G*″ > *G*′, *G*′~*ω*^2^, and *G*″~*ω* when *ω* → 0 for concentrated solutions (Figure 12c). In contrast, solutions of the terpolymer demonstrate anomalous gel-like behavior (*G*′ ≈ *const*) with its low content, which gradually fades as its concentration increases, transitioning to the typical viscoelastic response of a polymer solution (Figure 12d).

**Figure 13:**
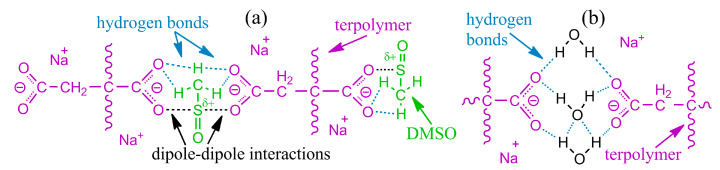
Interactions between acrylonitrile/sodium itaconate copolymer macromolecules in anhydrous DMSO (**a**) or the same solution in the presence of water (**b**) (adapted from [121]).

Thus, weak specific intermolecular interactions exist in terpolymer solutions, but manifest themselves only at low polymer concentrations, i.e., in the absence of macromolecular entanglements. Elasticity and yield stress in dilute solutions arise from supramolecular interactions of polymer chains through solvent molecules, i.e., via weak hydrogen bonds between the carbonyl oxygens of the polymer’s itaconic groups and the methyl hydrogens of DMSO in the presence of parallel ion–dipole and dipole–dipole interactions between oxygens of partially dissociated carboxylate groups and DMSO’s sulfurs (Figure 13a). In other words, DMSO molecules act as notional bridges, connecting dissolved polymer chains through two itaconic groups and forming an elastic supramolecular network manifesting itself rheologically: the specific strength of the supramolecular network and the distance between its nodes correlate with the yield stress of gel-like solutions and their storage moduli at low frequencies, respectively. Note that there is no supramolecular binding of chains and anomalous rheological behavior for solutions of the terpolymer in DMF or an aqueous solution of sodium thiocyanate. In addition, there is no structure formation of solutions of the homopolymer or itaconate-free copolymers in DMSO or other solvents [120,121,122,123,124] (except for copolymer solutions in *N*-methylmorpholine *N*-oxide, structuring on cooling [125]).

### 4.2. Microscopic Phase Separation of a Random Copolymer’s Concentrated Solution

**Figure 14:**
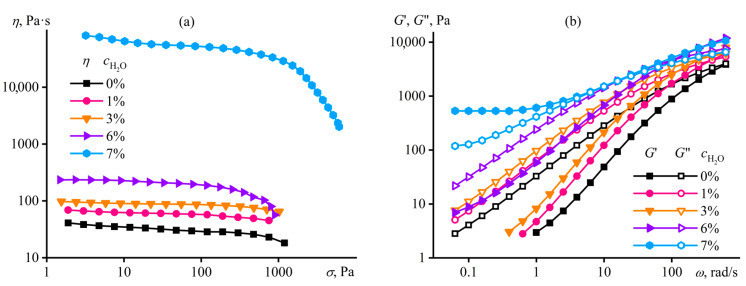
Viscosity vs. shear stress (**a**) and storage and loss moduli vs. angular frequency (**b**) for 18% solutions of acrylonitrile terpolymer in water-containing DMSO at 20 °C. The legends indicate the water mass fraction in the solutions (adapted from [120,121]).

Macromolecular entanglements conceal their weak intermolecular interactions, as a result of which the supramolecular structuring of the acrylonitrile copolymer manifests itself only in dilute solutions. This phenomenon arises due to the negligible contribution of non-covalent interactions to the total viscoelasticity of the concentrated systems and the dominance of macromolecular entanglements in determining their rheological properties, possibly due to the greater specific density or/and longer relaxation time (a time of existence) of chain entanglements than supramolecular contacts. However, the situation changes when making the macromolecular interactions stronger, e.g., by strengthening interchain hydrogen bonds through the addition of water to the polymer solution. In this case, gel-like behavior manifests even in concentrated solutions, but only at a specific content of the gel-forming agent—water [99,120,121]. The step-like addition of water to the concentrated solution of the acrylonitrile terpolymer elevates its viscosity (Figure 14a) because of an increase in the solution’s glass transition temperature, which gradually approaches the test temperature. At last, a small increment in the water mass fraction from 6% to 7% suddenly raises the viscosity by three decimal orders of magnitude, resulting in the gel formation. Frequency dependencies of storage and loss moduli confirm the gelation (Figure 14b). A small amount of water (up to 6%) increases the values of the moduli without qualitatively changing the pattern of their frequency dependencies. In contrast, the critical water content of 7% fundamentally changes their shapes: *G*′ ≈ *const* and *G*′ > *G*″ at low angular frequencies, indicating the formation of a spatial network based on non-covalent interactions and manifested at long observation times. This behavior is essential to understand and predict while producing membranes and fibers from polymer solutions when phase separation through the gelation stage slows down the structural rearrangement and changes the morphology of the shaped products.

In this case, the dominance of strong hydrogen bonds leads to the binding of macromolecules through molecules of the formal cosolvent (water) and its non-covalent interactions with itaconic groups of different chains (Figure 13b). Note that the addition of critical water concentration to the homopolymer solution causes macroscopic phase separation rather than gel formation. In other words, the strong structuring of the polymer solution with a gel formation is also specific, i.e., it occurs through specific non-covalent interactions. In addition, these interactions and the resulting gel formation do not suppress the dynamics of macromolecular chains or the topological nature of their entanglements: an increase in the frequency of external mechanical action elevates the storage and loss moduli, just like for an ordinary polymer solution (Figure 14b). The split in the frequency dependencies into the sections of the dominance of supramolecular interactions (at low frequencies) and macromolecular entanglements (at high ones) is due to the different lifetimes of these contacts: hydrogen bonds are longer-lived contacts (with a longer relaxation time) than macromolecular entanglements. Moreover, strong hydrogen bonds have a longer lifetime [126] than weaker non-covalent bonds in anhydrous systems (shown in Figure 13), which are rheologically visible only in dilute solutions.

**Figure 15:**
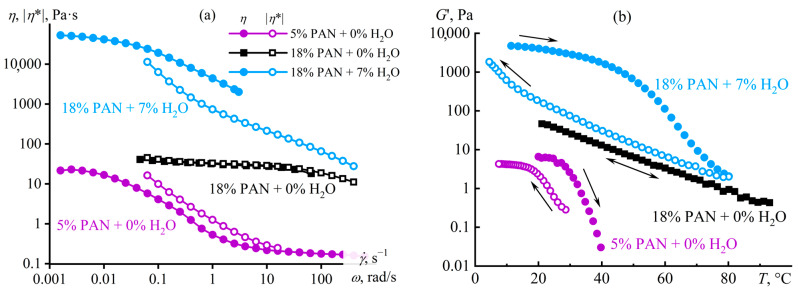
Steady-state viscosity and complex viscosity (**a**) at 20 °C as functions of shear rate and angular frequency, respectively, and the storage modulus (**b**) as a function of temperature during heating or cooling (directions indicated by arrows) for solutions of the acrylonitrile terpolymer in DMSO. The terpolymer mass fraction and water presence are near the curves (adapted from [120,121]).

Macromolecular entanglements and supramolecular interactions initiate viscoelasticity and non-Newtonian behavior, but of different natures [120]. In dilute solutions of the terpolymer, the steady-state viscosity is lower than the complex viscosity (5% PAN, Figure 15a). Continuous shear leads to the unfolding of polymer chains, reducing the density of their entanglements and decreasing the steady-state viscosity with the breakdown of interchain supramolecular contacts whose relaxation time exceeds that of topological entanglements (otherwise, the supramolecular binding would not manifest itself through a low-frequency plateau of the storage modulus in Figure 12d). Small-amplitude oscillatory shear does not cause structural changes, but models the dynamics of chains at different frequencies and rates from the standpoint of their relaxation. In a dilute solution, the contributions of supramolecular interactions and the topological entanglements initiated by them to total viscoelasticity are comparable. However, supramolecular interactions are broken or unbroken under continuous or small-amplitude shear, respectively, resulting in a lower steady-state viscosity relative to the complex viscosity. An increase in the polymer concentration extends the relaxation time of topological entanglements, and, as a result, supramolecular interactions do not manifest themselves because of the negligible contribution to the total viscoelasticity: the steady-state viscosity of the water-free concentrated terpolymer solution precisely coincides with its complex viscosity at similar shear rates and angular frequencies, indicating shear thinning as a consequence of elasticity caused by macromolecular entanglements (18% PAN, Figure 15a). The critical water content and the gelation of the solution increase the steady-state and complex viscosities, and the first becomes higher than the second. The cause of the latter is the nature of the gel formation—a microscopic phase separation with the appearance of a polymer-rich continuous phase, its glass transition, and very high viscosity, making the macroscopic phase separation infinitely long [127]. In turn, the steady-state viscosity depends on the resistance to flow exerted by the high-viscous glassy phase, while the complex viscosity is an average between the viscosities of coexisting phases, including the low-viscous solvent-rich phase, and, therefore, is lower.

The different natures of gel formation in dilute and water-containing solutions are also evident in the investigation of thermo-rheological properties [121]. The temperature dependence of the storage modulus of the water-free concentrated terpolymer solution is invariant, just like for a usual polymer solution (Figure 15b). On the contrary, a hysteresis of the rheo-curves is specific for the dilute solution because of the prolonged destruction/reconstruction of supramolecular contacts with an increase/decrease in temperature, and these contacts entirely determine the solution’s elasticity that disappears upon heating. In contrast, the elasticity of the water-containing concentrated solution does not disappear upon heating thanks to the preservation of macromolecular entanglements, but reduces by ten-fold nonetheless due to the loss of the gel-like state. Cooling causes the concentrated solution to transition back into the gel state, and the hysteresis of the storage modulus is much more pronounced than in the case of the dilute solution, as the destruction/reconstruction of supramolecular contacts takes much less time than the homogenization/phase separation of the concentrated system with the formation of the glass-state phase.

**Figure 16:**
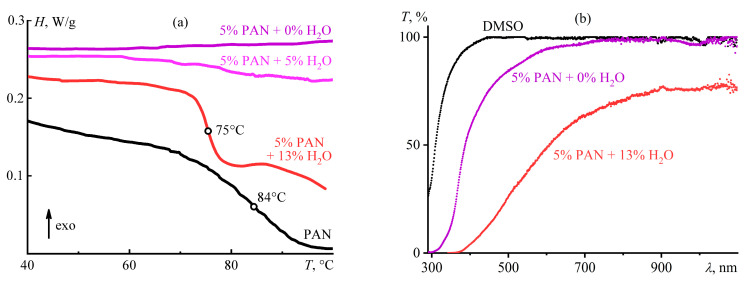
Thermograms of acrylonitrile terpolymer (PAN) and its 5% solutions in DMSO with different water mass fractions (**a**) and optical transparency of these solutions compared to DMSO at 20 °C (**b**) (adapted from [99,120]).

The water initiates microscopic phase separation and glass transition of dilute terpolymer solutions besides the concentrated ones, causing an increase in their stiffness (*G*′) and yield stress (*σ*_Y_). In all cases, differential scanning calorimetry and light scattering confirm the microscopic phase separation and the glass transition of the emerging phase [99,120]. An anhydrous solution or a solution containing a small amount of water does not exhibit temperature transitions upon heating, unlike a water-induced gel whose sudden change in heat capacity at 75 °C indicates glass transition (Figure 16a). The original terpolymer has a higher glass transition temperature, *T*_g_ = 84 °C, indicating the weak effect of water acting as a plasticizer of polyacrylonitrile. In addition, the anhydrous solutions hardly absorb visible light, unlike the hydrated gel (Figure 16b), whose light scattering indicates the microheterogeneity of its structure due to microscopic phase separation (many polymer gels fundamentally exhibit nanostructural heterogeneity as cross-linking irregularities and topological defects [128,129], but here and below refer to the phase heterogeneity).

### 4.3. Microscopic Phase Separation of a Homopolymer Solution

**Figure 17:**
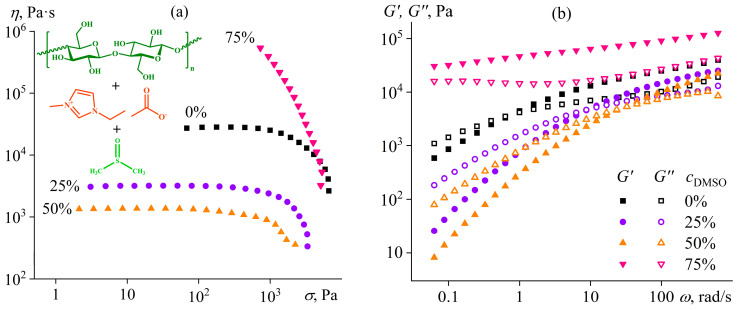
Viscosity as a function of shear stress (**a**) and storage and loss moduli as functions of angular frequency (**b**) at 25 °C for 14% cellulose solutions in [EMIM]Ac containing DMSO whose mass fraction in the solvent is near the curves or in the legend (adapted from [130]).

Water-containing gels of acrylonitrile terpolymer exist in a narrow range of the water mass fraction whose increase causes macroscopic phase separation, similar to the addition of water to acrylonitrile homopolymer solutions. Nevertheless, gel formation due to microscopic phase separation is possible in homopolymer solutions, e.g., cellulose in an ionic liquid—1-ethyl-3-methylimidazolium acetate ([EMIM]Ac)—with the addition of a nominal cosolvent [130]. Many ionic liquids can disrupt the intermolecular hydrogen bonds of cellulose and dissolve it [131,132]. However, their drawback is their high viscosity, which translates into high viscosity of the resulting polymer solutions, complicating their processing and initiating efforts to find low-viscosity cosolvents [133]. For cellulose, any polar aprotic solvent can be a cosolvent, for example, DMSO. It is a nominal cosolvent since it does not dissolve cellulose as an individual solvent. However, DMSO combined with [EMIM]Ac accelerates cellulose dissolution and reduces the viscosity of the resulting solutions by more than an order of magnitude: from 28,000 Pa·s to 1360 Pa·s at a cellulose mass fraction of 14 wt% and 25 °C, but when the DMSO content is not more than 50% in the solvent composition (Figure 17a) [130]. A DMSO mass fraction of 75% causes a multiple increase in viscosity—a gel formation, evidenced by the frequency dependencies of the storage and loss moduli (Figure 17b). A cellulose solution in pure ionic liquid or containing up to 50% DMSO shows no structure formation, as the rheo-curves are typical for a polymer solution (*G*′~*ω*^2^ and *G*″~*ω* when *ω →* 0). In contrast, both moduli are almost independent of frequency at 75% DMSO content, and the storage modulus exceeds the loss modulus. This behavior is typical for a gel [79].

**Figure 18:**
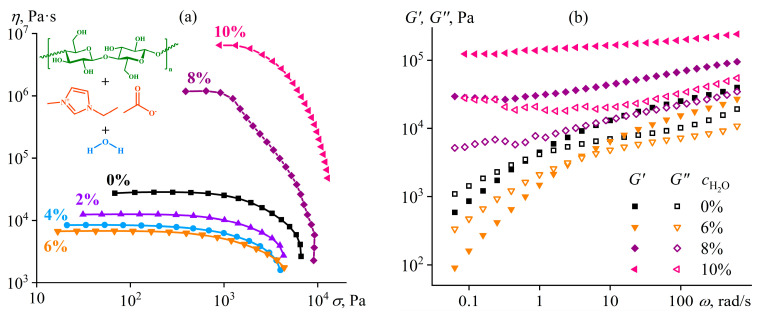
Viscosity vs. shear stress (**a**) and storage and loss moduli vs. angular frequency (**b**) for 14% cellulose solutions at 25 °C in [EMIM]Ac containing water whose mass fraction is near the curves or in the legend (adapted from [130]).

Polar protonic liquids can also be nominal cosolvents, e.g., alcohols and even water [130]. Extra water precipitates cellulose from its solution. However, water in small concentrations (up to 6% at a 14% cellulose content) dramatically reduces the viscosity of the cellulose solution in [EMIM]Ac (down to 6750 Pa·s, Figure 18a) without its structuring, judging by the Maxwell-like character of the frequency dependencies of the storage and loss moduli (Figure 18b). The primary reason for the viscosity reduction is the decrease in the viscosity of the ionic liquid (126 mPa·s) due to its dilution with less viscous water (0.89 mPa·s). Note that water, even in low concentrations, elevated the viscosity of acrylonitrile copolymer solutions (Figure 14a). In that case, the viscosities of DMSO (a solvent) and water (a non-solvent) are comparable: 2.0 mPa·s and 0.89 mPa·s. However, water worsens the thermodynamic quality of the resultant solvent, raising the solution’s glass transition temperature (Figure 16a) and thus increasing its viscosity.

An increase in the water content to the critical concentration (8%) leads to multiple rises in the viscosity of the cellulose solution (Figure 18a) and its gel formation (Figure 18b, *G*′ ≈ *const* and *G*′ > *G*″) because of microscopic phase separation [130]. The resulting gel exists in a limited concentration range of water. An increase in the water mass fraction to 10% elevates the gel’s stiffness and effective viscosity because the polymer concentrates in one of the coexisting continuous microscopic phases. However, the higher water content initiates macroscopic phase separation and cellulose precipitation.

In both the cases of water and DMSO, the appearance of specific interactions (hydrogen bonds) between cellulose macromolecules and their subsequent structuring results from a decrease in the concentration of the ionic liquid’s anions relative to the hydroxyl groups of cellulose. Upon DMSO addition, the concentration of the anions decreases proportionally to the dilution. In contrast, water molecules form hydrogen bonds with the anions besides the ionic liquid’s dilution, making them notionally inaccessible for interacting with cellulose macromolecules and thus leading to gel formation at a significantly lower mass fraction of water than DMSO.

### 4.4. Macromolecular Association and Mesophase Structuring of a Polyelectrolyte Solution

**Figure 19:**
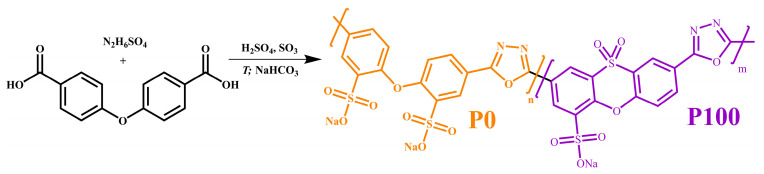
Synthesis scheme for sodium salts of poly(1,3,4-oxadiazole-2,5-diyl-3-sulfo-1,4-phenyleneoxy-2-sulfo-1,4-phenylene) (P0), poly(1,3,4-oxadiazole-2,5-diyl-6-sulfo-10,10-dioxophenoxathiine-2,8-diyl) (P100), and their copolymers (P25, P50, and P75).

The mentioned polymers of simple or complex structures could dissolve in individual solvents. However, rigid macromolecular chains may lead to solubility only in aggressive media, such as concentrated inorganic acids [134,135], but complex solvent mixtures can be an alternative. A one-pot synthesis in oleum allows for sulfonated copolymers of 1,3,4-oxadiazole, diphenyl ether, and 10,10-dioxophenoxathiine to be obtained (Figure 19) [136,137]. In this case, variants of pre-sulfonation of the initial 4,4′-oxybis(benzoic acid) result in statistical copolymers containing fragments of oxadiazole and diphenyl ether (P0, without prior sulfonation); oxadiazole and dioxophenoxathiine (P100); or their combinations (P25, P50, and P75), with comparable molecular weights of 150–180 kDa and sodium salt form due to neutralization of H_2_SO_4_ with NaHCO_3_ solution. The copolymers aim to be thermally stable ion- or proton-conducting materials and superabsorbents. However, polymer processing via sulfuric acid solutions is inconvenient, demanding new alternative non-corrosive complex solvents, e.g., equivolume mixtures of DMSO and formamide (FA) or DMSO, FA, and water.

**Figure 20:**
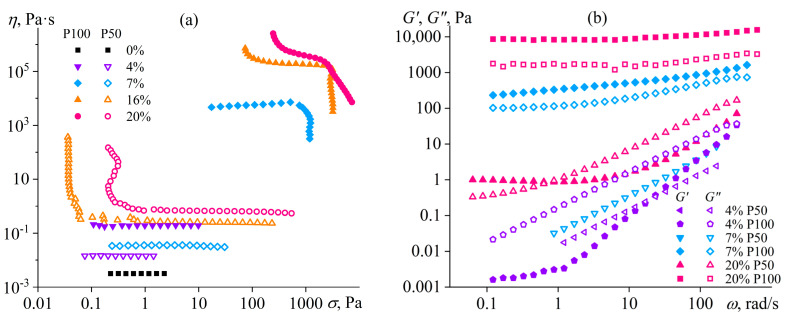
Viscosity as a function of shear stress (**a**) and storage and loss moduli as functions of angular frequency (**b**) for solutions of oxadiazole copolymers in an equivolume DMSO/FA mixture at 25 °C. Copolymers P0 and P25 are insoluble (adapted from [137]).

**Figure 21:**
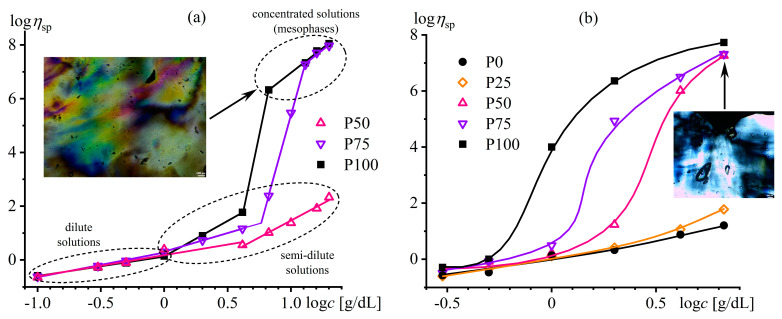
Specific viscosity of oxadiazole copolymers in an equivolume mixture of DMSO/FA (**a**) or DMSO/FA/water (**b**) at 25 °C. The insets show microphotographs of mesophases in crossed polarizers (3.5× magnification) (adapted from [137]).

The mix of FA and DMSO dissolves only copolymers containing no more than half of the disulfonated diphenyl ether fragments (P100, P75, P50) [137]. The equimolar copolymer (P50) forms both dilute (*c* < 16%) and semi-dilute solutions, and the latter structuralizes because of macromolecular associations, having a low yield stress (*σ*_Y_ < 1 Pa, Figure 20a) and demonstrating a prevalence of elasticity at long observation times (*G*′ > *G*″ when *ω* → 0, Figure 20b). Copolymers with a lower content of disulfonated fragments (e.g., P100) can additionally form concentrated solutions (*c* ≥ 7%) characterized by gel-like behavior (*G*′ ≈ *const* and *G*′ > *G*″, Figure 20b) and a simultaneous mesophase state (the inset in Figure 21a). The concentration dependencies of specific viscosity (Figure 21a) allow for detecting three concentration ranges: dilute (*η*_sp_~*c*^0.72–0.84^), semi-dilute (*η*_sp_~*c*^1.4–2.6^), and concentrated solutions (*η*_sp_~*c*^3.7^). The exponent for the dilute solutions is intermediate between those for solutions of neutral macromolecules and polyelectrolytes without entanglements (*c*^1.0–2.0^ and *c*^0.5–1.5^, respectively [138,139]), indicating partial dissociation of sulfonate groups. Partial dissociation also reflects itself in the intermediate exponents for the semi-dilute solutions (should be *c*^3.9^ and *c*^1.5^ for neutral and charged entangled macromolecules in a good solvent, respectively [138]) but is suppressed in the concentrated solutions.

**Figure 22:**
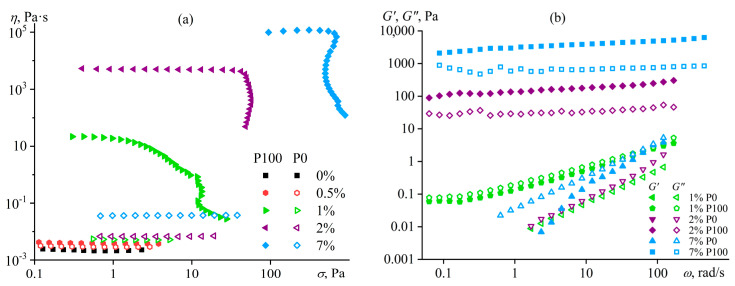
Viscosity as a function of shear stress (**a**) and storage and loss moduli as functions of angular frequency (**b**) for polyoxadiazole solutions in an equivolume DMSO/FA/water mixture at 25 °C (adapted from [137]).

The addition of water to the complex solvent allows for dissolving copolymers with high sulfonate content (P0, P25), but the gelation of the less sulfonated copolymers occurs at concentrations lower by 3–4 times. The least sulfonated polyoxadiazole P100 forms a semi-dilute structuralized solution at a mass fraction of 1%, exhibiting shear-thinning behavior (Figure 22a) and anomalous dependencies of the storage and loss moduli (Figure 22b). A higher P100 concentration of 2–7% forms a gel with an extended region of low-shear viscosity and a high yield stress (*σ*_Y_ > 50 Pa). In contrast, the most sulfonated copolymer P0 at a content of even 7% forms only a semi-dilute solution with ordinary Maxwell behavior. The concentration dependencies of the specific viscosity of the copolymers’ solutions vividly reflect the effect of their structure (sulfonate group content) on the transition between solution states and mesophase formation (Figure 21b), allowing for the solvent composition and polyoxadiazole mass fraction to be selected for forming ion-conducting films.

### 4.5. Structuring of Polymer Solution Due to Conformational Transition

**Figure 23:**
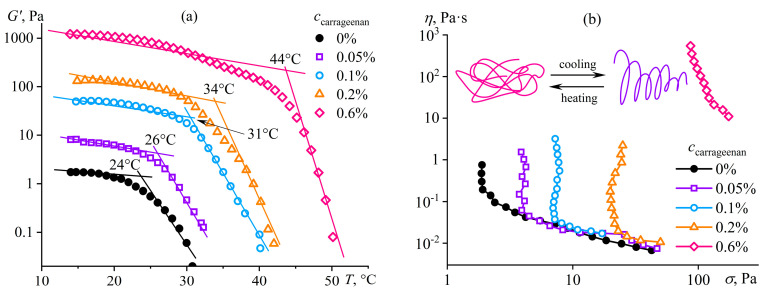
Storage modulus vs. temperature at an angular frequency of 6.28 rad/s (**a**) and viscosity vs. shear stress at 14 °C (**b**) for gelatin hydrogels (100 kDa, 1 wt%) containing *κ*-carrageenan (680 kDa), whose mass fraction is in the legends (adapted from [145]).

**Figure 24:**
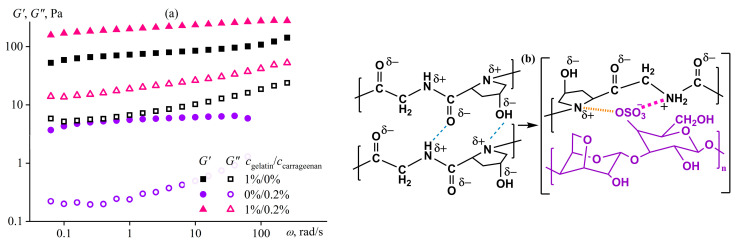
Storage and loss moduli versus angular frequency for gelatin, *κ*-carrageenan, and their combined hydrogels at 14 °C (**a**) and the transformation of intermolecular interactions of gelatin macromolecules in the presence of *κ*-carrageenan with an example of glycine and hydroxyproline units (**b**). Dashed lines represent hydrogen, ion–dipole, and ionic bonds (adapted from [145]).

In addition to solvent composition and polymer concentration, temperature also affects phase equilibrium in polymer solutions and, as a consequence, their phase state and rheology. Most often, structure formation happens upon a decrease in temperature because of a weakening of polymer/solvent van der Waals interactions relative to their separate interactions due to a rise in differences in cohesive energies [140]. Two principal variants are possible. The first is a change in the conformation of macromolecules due to the strengthening of intra- and inter-macromolecular non-covalent bonds while retaining the homogeneous state of the solution. The second is a decrease in the polymer solubility with phase separation, hindered by the glassy or rubbery state of the resulting polymer-rich (co)continuous phase, having structural elements of microscopic sizes. In the case of sulfonated polyoxadiazoles as polyelectrolytes, the transition of their macromolecules into a more stretched rod-like conformation [141] with an increase in their concentration most likely accompanies the formation of a mesophase. This transition, however, does not reduce the viscosity because of the strong interchain interactions and contrasts with the drop in viscosity of rigid-chain polymer solutions passing into the anisotropic state [117]. An example of a conformational transition due to cooling with the preservation of an isotropic state is the gelation of gelatin solutions whose macromolecules, as random coils, change their shapes to triple helix due to the strengthening of intermolecular hydrogen bonds [142]. The strength and stiffness of the resulting gel depend on the dissociation energy (*E*_D_) and density of intermolecular bonds, which can be reinforced by replacing part of the hydrogen bonds (*E*_D_ is within 3–46 kJ/mol [143]) with ion–dipole interactions (*E*_D_ ≈ 50–200 kJ/mol [144]) by adding the dissociating polysaccharide—*κ*-carrageenan. Cooling causes the transformation of the inelastic low-viscosity solution of pure or carrageenan-containing gelatin into a gel [145]. Moreover, the points of elasticity arising and gel formation shift towards higher temperatures with the addition of the polysaccharide (Figure 23a). The gel’s yield stress (Figure 23b) and stiffness increase similarly (Figure 24a). Gelatin and carrageenan exhibit synergy in joint gel formation, as the storage modulus of the composite gel (227 Pa) exceeds the sum of the moduli of pure gelatin and carrageenan gels (82 Pa and 6 Pa, respectively) at the same concentrations by 2.5 times. In other words, it is possible to produce a food gel with the same consistency but with significantly lower raw material consumption thanks to the synergy of the two biopolymers. In this case, the synergy is due to the dissociation of the carrageenan’s sulfate groups forming ion–dipole bonds with gelatin macromolecules and the protonation of the gelatin’s amide groups with an assemble of a polyelectrolyte complex, i.e., to the replacement of hydrogen bonds with stronger ones (Figure 24b).

### 4.6. Microscopic Phase Separation during Cooling of a Polymer Solution

**Figure 25:**
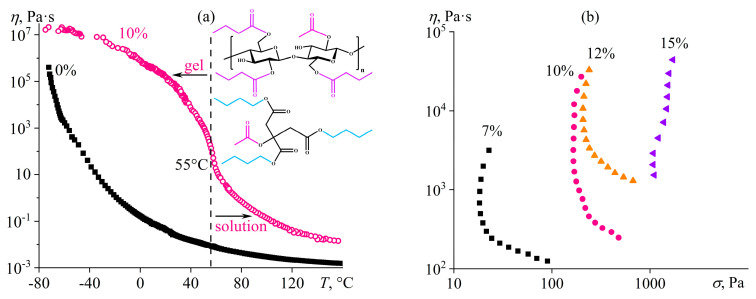
Viscosity versus temperature at a shear rate of 10 s^−1^ (**a**) or versus shear stress at 25 °C (**b**) for acetyl tributyl citrate (ATBC) containing cellulose acetobutyrate (CAB), whose mass fraction is near the curves (adapted from [146]). The viscosity of the pure ATBC grows smoothly upon cooling, whereas the viscosity of the CAB/ATBC solution rises in a step-like manner at 55 °C because of gel formation (**a**) manifesting itself in a yield stress behavior, where the yield stress value becomes higher with an increase in the CAB mass fraction (**b**).

The cooling of gelatin and *κ*-carrageenan solutions does not induce microscopic phase separation, which occurs during the gelation of cellulose or acrylonitrile copolymer solutions under the action of a cosolvent or precipitant. However, microscopic phase separation of a polymer solution in a single solvent can happen when lowering the temperature. An example is cellulose acetate butyrate (CAB, 2% acetyl, 47% butyrate, and 4.8% hydroxyl groups, 68 kDa) in a solvent of a similar structure—acetyl tributyl citrate (ATBC) [146]. Cooling the CAB solution below 55 °C induces a significant increase in viscosity not observed for the pure solvent, which does not crystallize until −80 °C (Figure 25a). The increase in viscosity is followed by clouding due to the microscopic phase separation and the appearance of the yield stress, which grows with the rising content of CAB (Figure 25b). A characteristic feature of CAB gels is the rubbery state of their polymer-rich phase, unlike the microheterogeneous gels of cellulose or acrylonitrile copolymer, which are in a glassy state. The difference in states manifests itself in the approximately tenfold lower yield stress of rubbery-like gels and their ability to flow at shear stresses exceeding the yield stress (given co-continuous states of both phases, i.e., at moderate polymer concentrations). The flow mechanism involves the continuous breakage of structural elements of the polymer-rich continuous phase with their subsequent thixotropic self-healing due to the mobility of macromolecules, which is absent in glassy gels whose structure and rheological properties do not fully recover after exposure to high shear stresses.

**Figure 26:**
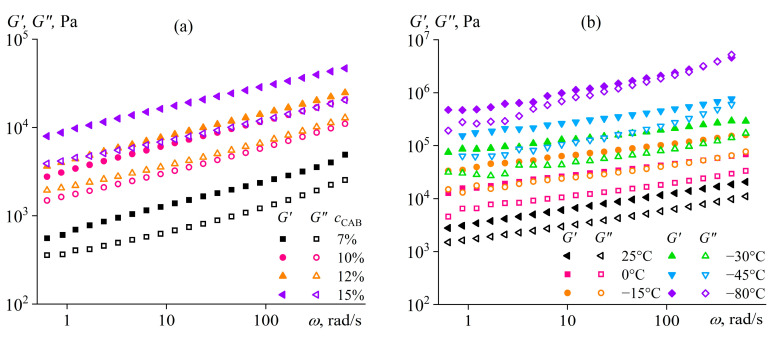
Frequency dependencies of storage and loss moduli for CAB/ATBC gels of different compositions at 25 °C (**a**) and at different temperatures and a 10% CAB mass fraction (**b**) (adapted from [146]). Higher CAB content (**a**) and cooling (**b**) elevate gels’ stiffness, but only a temperature as low as −80 °C and high applied frequencies cause their notable mechanical glass transition, i.e., an increase in the moduli upon a rise in angular frequency, indicating a rubbery-like state of the gels at higher temperatures.

The storage modulus of the rubbery-like CAB gel exceeds the loss modulus (Figure 26a). However, the storage modulus’s dependence on the angular frequency is 2–4 times more intensive (*G*′~*ω*^0.26–0.30^) than in the case of gels caused by glass transition, formation of a mesophase, or conformational coil/helix transition (*G*′~*ω*^0.06–0.16^). The feature results from the polydispersity of cellulose macromolecules (*Đ*_CAB_ = 3.3), which mix the areas of terminal viscoelasticity (*G*′~*ω*^2.0^) and rubbery-like elasticity (*G*′ ≈ *const*) of different-length chains but almost do not affect their glassy state or phase/conformational transitions. An increase in the CAB content raises the gel’s storage and loss moduli because of the higher volume of the polymer-rich phase. A temperature decrease acts similarly, but only strong cooling to −80 °C makes the mechanical glass transition of the CAB gel at high frequencies noticeable (Figure 26b). Cooling enriches and depletes the two coexisting phases with the polymer due to the reduction in the CAB/ATBC mutual solubility, which nevertheless does not disappear, allowing for the gel to be used at very low temperatures, e.g., as a lubricating grease.

### 4.7. Microscopic Phase Separation during Heating and Intense Shear of a Polymer Solution

**Figure 27:**
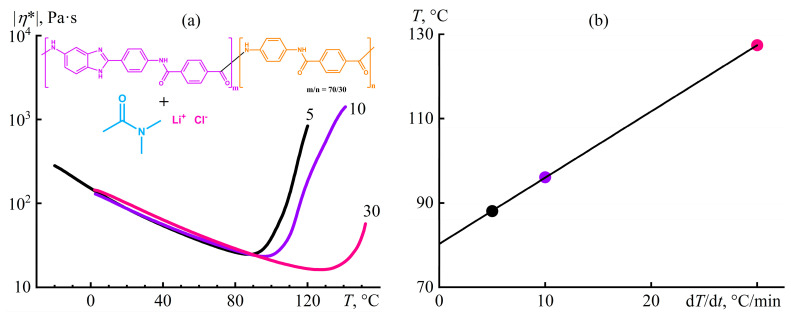
Complex viscosity of a 5% solution of benzimidazole copolymer in DMA as a function of temperature at an angular frequency of 6.28 rad/s, a strain amplitude of 10%, and the heating rate indicated in °C/min near the curves (**a**), as well as the temperature of the phase separation of this solution as a function of the heating rate (**b**) (adapted from [100]).

Gel formation under an increase in temperature occurs less frequently and usually results from the decay of polymer/solvent hydrogen bonds [140,147,148]. An example is solutions of polybenzimidazole/poly(*p*-phenylene-terephthalamide)-based copolymers in *N*,*N*-dimethylacetamide (DMA, containing 3% LiCl to improve solubility) [100,149], which have found application in the production of aramid fibers [150]. The complex viscosity of a 5% solution of poly(benzimidazole-2,6-diyl-iminoterephthaloylimino-1,4-phenylene-*co*-iminoterephthaloylimino-1,4-phenylene) ([*η*]_DMA,20°C_ = 6.2 dL/g) gradually decreases upon heating following the acceleration in molecular thermal motion and the reduction in intermolecular interactions, passes through a minimum, and then sharply rises because of the microscopic phase separation with the gel formation (Figure 27a). The increase in the heating rate linearly raises the temperature of the phase separation (Figure 27b), indicating its kinetic nature. Extrapolation of the obtained straight line to the ordinate axis allows for the temperature of the phase transition under static quasi-equilibrium conditions (80.3 °C) to be determined, whereas the line’s slope presents its nominal duration (94 s).

**Figure 28:**
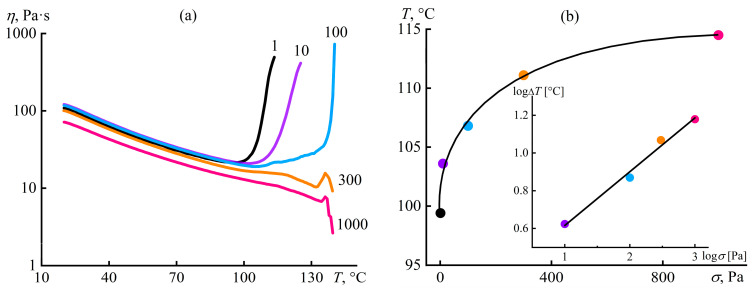
Viscosity of a 5% solution of benzimidazole copolymer in DMA as a function of temperature at a heating rate of 10 °C/min and a shear stress indicated in Pa near the curves (**a**), as well as the temperature of the phase separation of this solution as a function of the shear stress (**b**). The inset shows the increase in the phase separation temperature under shear action (adapted from [100]).

The measurement of the complex viscosity occurs at the low strain, i.e., without shear influence on the solution structure and phase equilibrium. Continuous shear at 1 Pa also does not shift the phase separation point (a viscosity minimum) in contrast to higher shear stresses (Figure 28a; *σ* = 300–1000 Pa causes wall slip). The increment in the transition temperature with increasing shear intensity is linear in log–log coordinates (Figure 28b), but only 1000 Pa decreases the viscosity over the entire temperature range, i.e., causes noticeable chain stretching and, hence, entropy reduction [151]. The entropy-free effect indicates the kinetic nature of the temperature shift due to the mixing and destruction of nucleation centers [152,153] when, for example, a shear stress of 1000 Pa raises the phase separation temperature by 14.5 °C, which is equivalent to an increase in the phase separation time from 94 s to 181 s [100].

### 4.8. Phase Separation Initiated by Intense Shear

**Figure 29:**
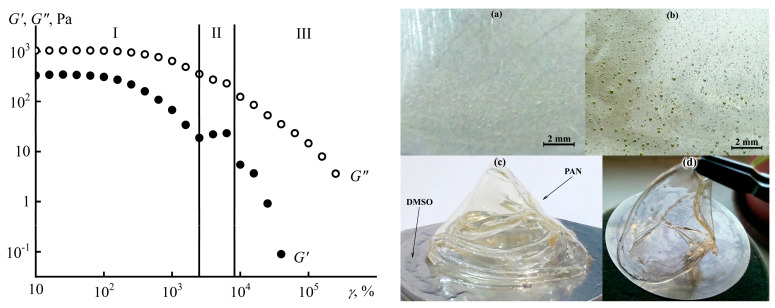
Storage and loss moduli as functions of strain amplitude for a 20% solution of acrylonitrile terpolymer in DMSO at an angular frequency of 6.28 rad/s and 20 °C, as well as photographs of the solution after testing with strains of 1600% ((**a**), zone I of macromolecular orientation), 4000% ((**b**), zone II of phase separation), and 250,000% ((**c**,**d**), zone III of wall slip) (adapted from [99]).

Nevertheless, intense shear may initiate the phase separation of a concentrated polymer solution due to thermodynamic reasons: chain stretching and entropy decrease [151,152,153], as in the case of the multiblock vinyl acetate/vinyl alcohol copolymer (see Figure 11b) or, for example, the acrylonitrile terpolymer (PAN) [99,121]. Continuous high-intensity shear of PAN solution in DMSO is impossible due to the Weissenberg effect, but high shear rates can result from applying large-amplitude oscillatory shear (Figure 29). An increase in the strain amplitude gradually reduces the storage and loss moduli of the solution due to chain stretching and a decrease in the density of macromolecular entanglements (zone I), maintaining its homogeneity (Figure 29a). However, a rise in the elasticity happens at *γ* ≈ 3000% (zone II), accompanied by the appearance of bubbles of polymer-depleted solvent in the solution (Figure 29b). Further strain growth above 10,000% causes an apparent decrease in the moduli (zone III) due to wall slip resulting from the solvent release on the sample becoming an elastic film because of macroscopic phase separation (Figure 29c,d). This effect of initiating (as for PAN) or delaying (as for the benzimidazole copolymer) phase separation under intense deformation is potentially exploitable in shaping polymer fibers, e.g., by mechanotropic spinning [154].

### 4.9. Element Sizes in Structuralized Polymer Solutions

Thus, the structure formation in polymer solutions may occur due to the macromolecular association because of the complex chain structure and specific intermolecular/supramolecular interactions, conformational transition, mesophase formation, or microscopic phase separation upon the addition of co-/non-solvents, temperature change, or intense shear up to macroscopic phase separation. The phase state of the structured system can be both homo- and microheterogeneous, and the rheological manifestation of the structure is solid-like behavior (*G*′ ≈ *const*). The magnitude of the storage modulus characterizes the average distance between the structural network nodes [81]. By simplifying the comparison of gels that have different chemical natures, this distance can be expressed through a polyethylene equivalent: *l* = (*ρRTl*_C–C_)/(*G*′*M*_m_), where *ρ* is the density, *R* is the universal gas constant, *T* is the absolute temperature, *l*_C–C_ is the length of the C–C bond (0.1535 nm), and *M*_m_ is the molecular weight of the methylene group, i.e., via a nominal distance between nodes, as if the gels were from polyethylene macromolecules. If *G*′ = 1000 Pa, then *l* = 25 µm, and the gel formation leads to microstructuring, i.e., to a system whose structural elements have micron-scale sizes. The highest storage modulus observed among the reviewed gels at moderate temperatures is about 10^5^ Pa (Figure 18b), corresponding to an *l* value as low as 0.25 µm. Moreover, the complication in chain structure, getting away from the methylene groups, reduces the distance between gel nodes by approximately as many times as the molecular weight of the new monomeric unit exceeds that of a polyethylene unit. In other words, the actual distance may be much smaller but still not in the decimal orders of magnitude, i.e., be of submicron scales rather than nanometer ones. Thus, the structuring of polymer systems due to non-covalent intermolecular interactions leads to the formation of micro- and submicrostructures, although nanoscale structures are nonetheless possible in melts of block and multiblock copolymers.

## 5. Specific Interactions in Polymer Dispersions

### 5.1. Structure Formation in a Dilute Mix of Particle Dispersion and Polymer Solution

**Figure 30:**
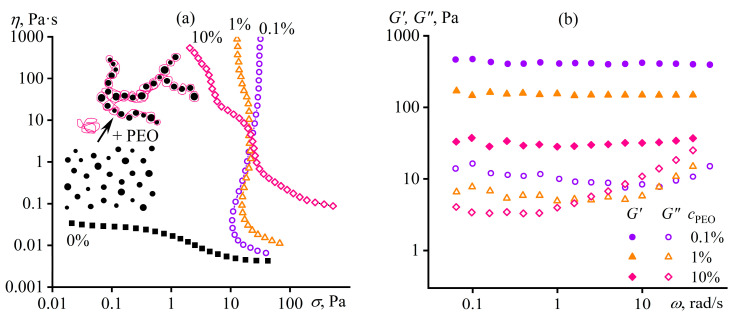
Viscosity as a function of shear stress (**a**) and storage and loss moduli as a function of angular frequency (**b**) for a 3 vol% dispersion of fumed silica (7 nm, 388 m^2^/g) in DMSO containing polyethylene oxide (40 kDa) whose mass fraction is indicated near the curves and in the legend. The SiO_2_ dispersion and 0.1–10% PEO solutions do not demonstrate viscoelasticity separately. *T* = 50 °C, preventing crystallization of PEO. The inset shows a schematic change in the structure of the SiO_2_ dispersion in the presence of PEO (adapted from [155]).

Another way of structuring a polymer medium is to introduce dispersed particles whose specific interactions among themselves and with macromolecular chains will reflect in the structure and rheological properties of the resulting polymer material, which is essential to understand and predict when producing polymer nanocomposites. For example, macromolecules of polyethylene oxide (PEO) in a dilute dispersion of silica nanoparticles in DMSO dramatically change the dispersion’s behavior from weak pseudoplasticity to expressed viscoplasticity (Figure 30a), which results from gel formation (*G*′ > *G*″, *G*′ ≈ *const*, Figure 30b) [155]. Since neither PEO molecules nor SiO_2_ nanoparticles individually form a spatial network in DMSO at their low concentrations, the sol–gel transition is due to the adsorption of macromolecules onto numerous particles, which binds them together into a single structural network (inset in Figure 30a). However, a rise in the PEO concentration from 0.1% to 1% and then to 10% reduces the gel’s yield stress and stiffness because of the increasing coverage of particles’ surfaces by macromolecules and the resulting decrease in the direct coagulation interparticle contacts. High shear stresses disrupt gel networks, and the viscosity of the SiO_2_/PEO dispersions tends towards the viscosity of PEO solutions without particles.

### 5.2. Structure Suppression in a Concentrated Mix of Particle Dispersion and Polymer Solution

**Figure 31:**
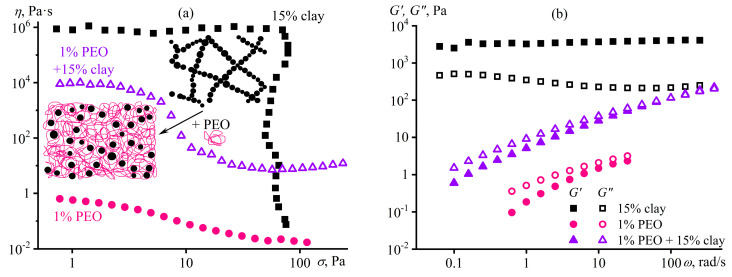
Viscosity as a function of shear stress (**a**) and storage and loss moduli as a function of angular frequency (**b**) for a 1% aqueous solution of polyethylene oxide (3 MDa), a 15% aqueous dispersion of bentonite (75 µm, 58 m^2^/g), and their combination at 25 °C. The inset shows a schematic change in the structure of the bentonite dispersion in the presence of PEO (adapted from [156]).

Small amounts of macromolecules do not change the behavior of concentrated dispersions, just as a small number of particles does not affect the properties of concentrated polymer solutions, unlike the combination of two concentrated systems (Figure 31) [156]. A dispersion containing 15% bentonite clay in water is a gel with a low-shear viscosity plateau, yield stress, and dominance of elastic behavior, whereas a 1% aqueous solution of ultra-high-molecular-weight PEO is a concentrated polymer solution exhibiting pseudoplasticity and viscoelasticity. Despite the low mass fraction of macromolecules, they entirely suppress the structuring of bentonite particles, as the mixed system resembles the original polymer solution, albeit with higher viscosity, more pronounced non-Newtonian behavior, and increased storage and loss moduli. The four-order rise in the viscosity corresponds to a nominal increase in the molecular weight of the polymer medium due to clay particles, since *η*~*c*[*η*]~*cM^α^* for polymer solutions where *α* ≈ 0.7 [57]. This rise indicates a structured state of the mixed system where macromolecules adsorbed on the same clay particles behave as a whole while these particles do not directly contact each other (Figure 31a), acting as bridges for PEO molecules and thus increasing their apparent molecular weight. In other words, the physical network of entangled macromolecules suppresses the formation of a coagulation network from clay particles, and the adsorption nodes of macromolecules participate in determining the relaxation properties of the resulting structured solution on par with the usual nodes of macromolecular entanglements.

**Figure 32:**
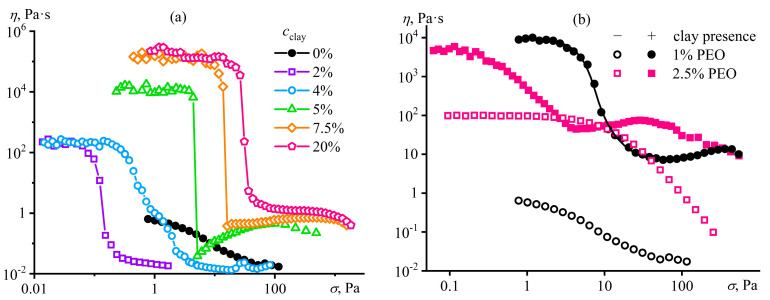
Viscosity as a function of shear stress at 25 °C for a 1% aqueous PEO solution containing different bentonite mass fractions (**a**) and a 15% aqueous bentonite dispersion containing different PEO mass fractions (**b**) in comparison with the initial bentonite-free PEO solutions (adapted from [156]).

The non-Newtonian behavior of the mixed system corresponds to nominal yield stress, which increases with the content of solid particles (Figure 32a) and matches the total strength of PEO/bentonite adsorption contacts. At high shear stresses, the viscosity of the mixtures containing 2–4% clay drops to that of the original PEO solution with macromolecules disentangled by intense shear. In contrast, the viscosity of more concentrated systems remains much higher at high shear stresses, indicating the maintenance of some adsorption contacts, possibly due to the intercalation of polymer chains into the interlayer space of clay tactoids. Furthermore, dispersions with 5–15% clay demonstrate dilatancy, which is absent in the original solutions (Figure 32b) and caused by the shear-induced temporary rise in the entanglement density of deformed chains whose permanent adsorption nodes obstruct their disentanglement and orientation. An increase in the PEO content from 1% to 2.5% does not change the low-shear viscosity of the mixed system, although the viscosity of the clay-free PEO solution goes up a hundredfold. This fact indicates the constancy of the total number of nodes in the physical network despite the increase in the concentration of macromolecules and, consequently, their entanglements, indirectly implying a reduction in the density of PEO/clay adsorption nodes. As a result, the viscosity of the mixture with 2.5% PEO first decreases and then increases at lower shear stresses than the one with 1% PEO due to a lower total strength of weak PEO/bentonite adsorption contacts and a higher density of macromolecular entanglements, respectively.

### 5.3. Effect of Dispersed Particles on the Structure and Rheology of Polymer Gels

**Figure 33:**
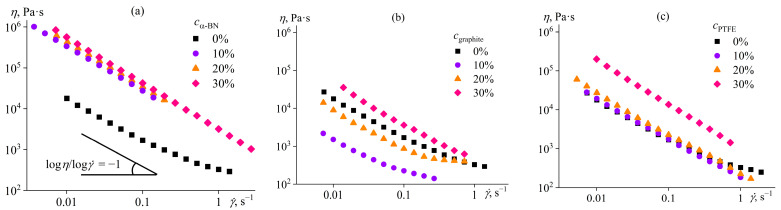

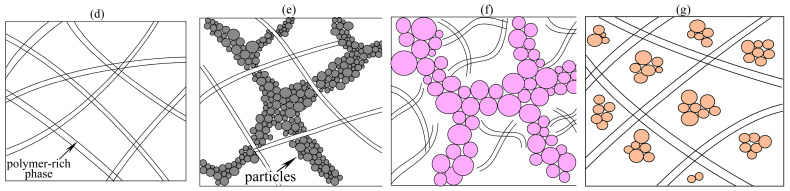
Viscosity versus the shear rate at 25 °C for 10% CAB/ATBC gels containing boron nitride (**a**), graphite (**b**), or PTFE (**c**) and a scheme of transforming the initial gel structure (**d**) with the formation of an additional particle network (**e**), destruction of the polymer network with the formation of a particle network (**f**), or the action of particles as inactive fillers (**g**) (adapted from [146]).

The solid particles influence the structure and rheological properties of microheterogeneous polymer gels, in which macromolecular entanglements are absent throughout the volume but concentrated in the polymer-rich phase (Figure 33d) [146]. Depending on the surface’s nature and the tendency of particles to form a structure, their synergistic combination, competition, or neutral coexistence with the polymer gel network is possible. The addition of 10% hexagonal boron nitride particles (1.6 μm) into 10% CAB gel in ATBC significantly increases its effective viscosity and yield stress (*σ*_Y_ = *η*$\dot{\gamma }$ when the slope of log*η*(log$\dot{\gamma }$) equals −1 over a wide range of shear rates; Figure 33a). A further increase in the particle mass fraction to 20–30% has little effect, indicating that BN particles are incorporated into the existing polymer gel network as either a co-continuous or joint network (Figure 33e) while their excess remains in a non-agglomerated state. In contrast, 10% graphite particles (2.0 μm) significantly reduce the viscosity and yield stress of the CAB gel, whereas their higher mass fractions increase these characteristics (Figure 33b). In this case, graphite first disrupts the polymer gel network by adsorbing CAB macromolecules and then forms the network from coagulated particles (Figure 33f). Polytetrafluoroethylene particles (PTFE, 0.53 μm) are inert and have little effect on the gel properties at 10–20% content (Figure 33c), serving as inactive fillers (Figure 33g). However, PTFE forms a network at a 30% concentration, changing the gel’s structure (Figure 33e,f) and increasing its strength (i.e., *σ*_Y_).

### 5.4. Effect of Dispersed Particles on Structure and Rheology of Polymer Melt

**Figure 34:**
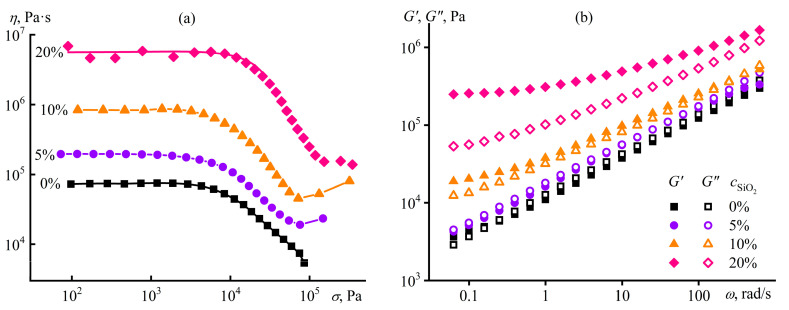
Viscosity versus shear stress (**a**) and storage and loss moduli versus angular frequency (**b**) at 20 °C for an adhesive mixture of poly(iso)butylenes (40% 4.5 kDa, 50% 51 kDa, and 10% 1.1 MDa) containing pyrogenic silica particles (20 nm, 175 m^2^/g) whose mass fraction is indicated near the curves or in the legend (adapted from [157]).

In the case of a homogeneous polymer medium containing uniform macromolecular entanglements within the whole volume, for example, polyisobutylene elastomer (PIB), the addition of SiO_2_ nanoparticles gradually increases its viscosity without the appearance of yield stress (Figure 34a) [157]. In this regard, the elastomer’s macromolecular entanglements suppress the structuring of nanoparticles, similar to the entanglements of the concentrated polymer solution (Figure 31). Moreover, a region of dilatant viscosity growth also appears at high shear stresses, associated with increased entanglement density of deformed chains, whose adsorption on nanoparticles hinders their orientation, similarly to the particle-containing concentrated polymer solutions (Figure 32). Interestingly, the nanoparticles cause the storage modulus to increase more intensively than the loss modulus (Figure 34b). As a result, the filled elastomer behaves like a gel at a 20% SiO_2_ content despite the absence of a yield stress. Macromolecular chains adsorbed on a single particle act as a whole, formally increasing the molecular weight of the polymer medium and converting it from a viscous liquid to a high-elastic rubbery-like relaxation state (a 70-fold rise in viscosity at 20% SiO_2_ corresponds to a formal increase in the molecular weight by 3.5 times since *η*~*M*^3.4^ [57]). In other words, nanoparticles act as temporary cross-links for the polymer matrix due to chain adsorption and macromolecular entanglements.

### 5.5. Effects of Medium’s Molecular Weight and Particles’ Size on the Rheology of Dispersions

**Figure 35:**
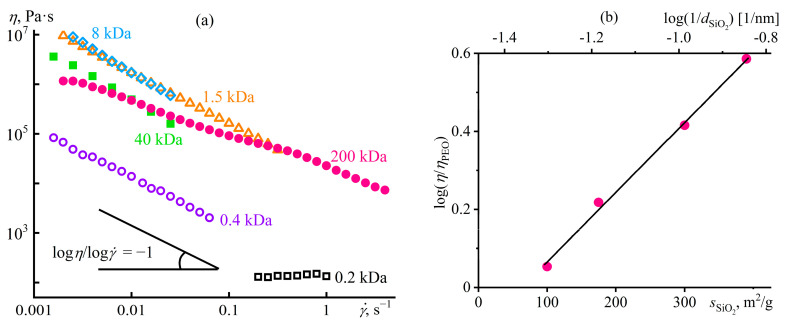
Viscosity versus shear rate for 7% SiO_2_ dispersions (7 nm, 388 m^2^/g) in PEO melts having different molecular weights (indicated near the curves) at 120 °C (**a**) and the relative viscosity of 3% SiO_2_ dispersions in PEO (400 Da) at 20 °C versus particles’ specific surface area and size (**b**) (adapted from [162]).

At high concentrations, dispersed particles structuralize in a low-molecular-weight medium because of contact interactions [158,159,160]. However, an increase in the molecular weight of the medium generates an entanglement network, which may affect the formation of a coagulation network from dispersed particles. Macromolecular entanglements arise at a critical molecular weight, e.g., at approximately 4 kDa for PEO [161]. The investigation of 7% SiO_2_ dispersions in the PEO melts shows that they form a gel when *M*_PEO_ is within about 0.4–40 kDa (see Figure 35a) [162]. A lower *M*_PEO_ (0.2 kDa) is insufficient for the adsorption of one PEO molecule onto different particles and their binding, whereas a higher *M*_PEO_ (200 kDa) suppresses the particles’ structure formation because of dense macromolecular entanglements. In this case, the gel’s effective viscosity and yield stress (*σ*_Y_ = *η*$\dot{\gamma }$) rise with increasing *M*_PEO_ from 0.4 kDa to 1.5 kDa, remain unchanged to 8 kDa, and then decrease when transitioning to 40 kDa. Since the Kuhn segment’s length for PEO is around 0.2 kDa (3–5 monomer units [163,164]), the gel formation of nanoparticles via adsorption of PEO molecules occurs at a molecular length twice as high (0.4 kDa), reaches the maximum for flexible oligomers and low-molecular-weight polymers (1.5–8 kDa), and then declines upon increasing the number of entanglements per one macromolecular chain (>8 kDa).

**Figure 36:**
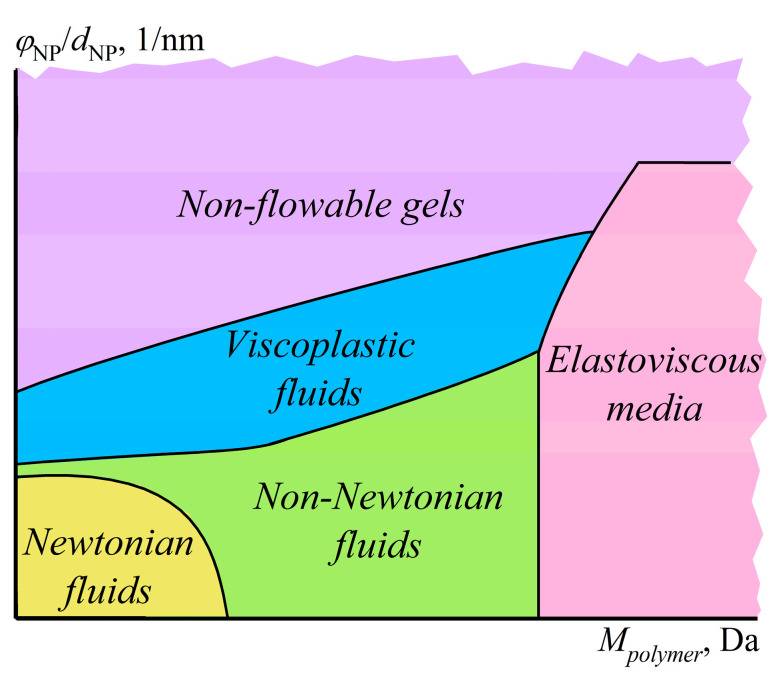
Schematic diagram of the rheological state for dispersions of particles in polymer media depending on their size and volume fraction and the polymer’s molecular weight (adapted from [162]).

The size of particles and, consequently, their specific surface area also influence rheological properties. Under equal conditions, the increase in the specific surface area of silica nanoparticles (*s*_SiO2_) exponentially elevates the apparent viscosity of their dilute dispersions in the PEO medium (Figure 35b). In addition, a decrease in the size of particles reduces their percolation threshold (to as low as 0.0036 vol% for 14 nm silver particles in water [165]) and the actual maximum packing density (e.g., to 5 vol% for 7 nm SiO_2_ particles in C_15_H_32_ [98]), allowing for a generalization of their rheological behavior in polymer media through a diagram (Figure 36). An increase in the volume fraction and a reduction in the size of the particles leads to a transition from Newtonian fluids to pseudo- and then viscoplastic systems (potentially also exhibiting the low-shear plateau viscosity and dilatancy), while growth in the polymer medium’s molecular weight causes a transition from Newtonian fluids to pseudoplastic and then elastoviscous high-elastic systems. The combination of macromolecules and particles results in superimposing two trajectories, which is valuable for obtaining polymer composites, which must have or conversely not have a structural network from filler particles.

### 5.6. Structuring of Dispersions of Polymer Particles

**Figure 37:**
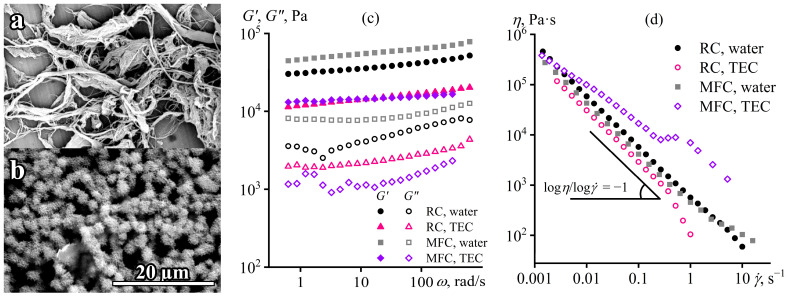
SEM images of (**a**) microfibrillated cellulose (MFC) and (**b**) regenerated cellulose (RC), (**c**) dependencies of storage and loss moduli on the angular frequency for their 3% hydro- and organogels, and (**d**) the viscosity curves of these gels (adapted from [168,169,170]).

However, the dispersed particles also may have a polymeric nature, as in the case of submicro- and nanoscale cellulose particles (co-called nanocellulose [166,167]). The most inexpensive is microfibrillated cellulose (MFC), having microfibers with a diameter of about 0.1–1 μm and a length of over 100 μm (Figure 37a) [168]. The particle shape is different for regenerated cellulose (RC)—amorphous spherical aggregates having sizes of around 2 μm and consisting of smaller particles with a diameter of about 120 nm (Figure 37b), obtained by precipitating cellulose from its dilute solutions, e.g., in a hot mixture of *N*-methylmorpholine *N*-oxide and DMSO (3/7 wt/wt) [169]. Both types of nanocellulose particles form a gel in water or organic polar liquids (e.g., triethyl citrate, TEC), as *G*′ > *G*″ and *G*′ ≈ *const* (Figure 37c), but hydrogels exhibit greater stiffness than organogels at equal mass fraction of cellulose particles, probably because of their better swelling in the aqueous medium and, thus, increased volume fraction [168,169,170].

The nanocellulose gels are viscoplastic, as their viscosity curves have a slope of −45° in log–log coordinates (Figure 37d). However, the hydrogels can flow at high shear rates of up to 10 s^−1^, whereas organogels demonstrate wall slip (viscosity curve slope < −45°) or dilatancy with subsequent wall slip at lower shear rates of around 0.3–0.4 s^−1^ for RC or MFC particles, respectively. Similarly to entangled macromolecules, there are entanglements between microfibrils, and the decrease in the effective viscosity with increasing shear rate is because of the orientation of microfibrils during the flow and the reduction in the density of their entanglements [168]. The rise in shear rate increases the friction within entanglement nodes, causing individual microfibrils to cease movement and transforming fluctuational nodes into permanent ones. This disentanglement stoppage increases the entanglement density in deformed microfibrils and, consequently, the effective viscosity of their dispersion. In other words, flexible entangled microfibrils tighten within some nodes, which start acting similarly to adsorption nodes in filled polymer systems (Figure 32 and Figure 34a), increasing the viscosity and impeding flow at high shear rates. There are no entanglements between the sphere-like RC particles, but the same shear rates cause increased friction between them in the TEC medium and the cessation of flow with the transition to wall slip [169]. In water, the interactions between cellulose particles are weaker due to their better solvation, and the effects of dilatancy and wall slip do not appear under comparable conditions.

**Figure 38:**
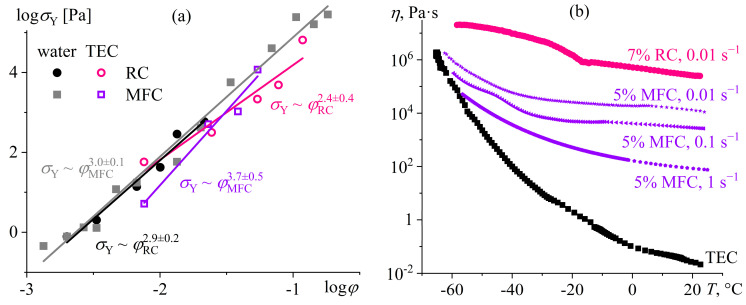
Yield stress of gels versus nanocellulose content (**a**) and temperature dependencies of viscosity for two organogels compared to pure TEC (**b**) (adapted from [168,169,170]).

The yield stress (*σ*_Y_ = *η*$\dot{\gamma }$) of gels corresponds to the strength of interparticle contacts. In the case of hydrogels, the dependence of *σ*_Y_(*φ*) does not depend on the type of nanocellulose: *σ*_Y_~*φ*^2.9–3.0^ (Figure 38a). In contrast, microfibrils increase the yield stress of gels in TEC more intensively than spherical particles (*σ*_Y_~*φ*^3.7^ and *φ*^2.4^, respectively). The rise in *σ*_Y_ with the content of spherical particles occurs due to the increase in the number of particles and their interparticle contacts. However, in the case of microfibril dispersions, the density of interfibrillar entanglements also elevates with the cellulose content, explaining the more intensive growth of their yield stress. The cellulose swelling is higher in water and the strength of interparticle interactions is lower, judging from the absence of pronounced dilatancy of the MFC dispersions, which levels out the morphological difference of the nanocellulose particles, and the dependencies *σ*_Y_(*φ*) appear to be similar.

The advantages of TEC include a low melting point (−55 °C), allowing for the use of nanocellulose organogels in extreme cold (Figure 38b). Temperature reduction increases the effective viscosity of gels as high as within three orders of magnitude, unlike the eight-order rise in viscosity for pure TEC. The viscoplastic behavior of gels remains intact since a tenfold increase in shear rate comparably reduces their effective viscosity, meaning that cooling raises the yield stress, which allows for low-temperature gels of the necessary consistency to be created by adjusting the content of nanocellulose—either spherical or microfibrillated, e.g., for use as biodegradable greases [168,169].

Cellulose microfibrils resemble flexible entangled macromolecules, while regenerated cellulose resemble macromolecular particles that nonetheless can dissolve and form melts while maintaining their complex architecture. The complex architecture will manifest itself in the rheological properties of polymer solutions and melts with enhancements when transitioning from rarely branched macromolecules to spherical ones [171,172,173].

## 6. Complex Chain Architecture and Its Manifestation in Rheological Properties

### 6.1. Reflection of Long-Chain Polymer Branching in Elasticity Arising at Flow

**Figure 39:**
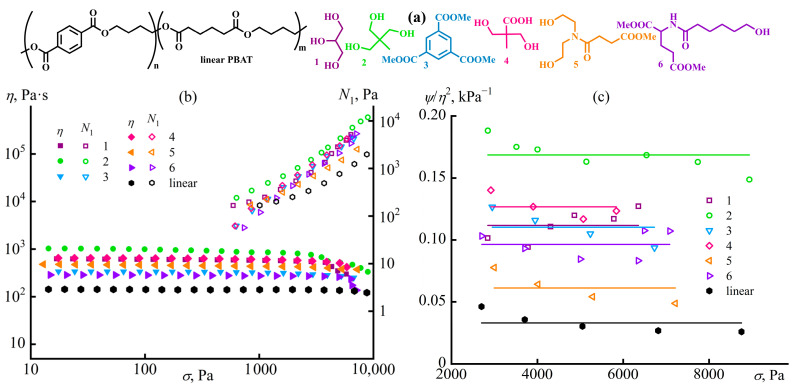
Structural formulas of PBAT and polyfunctional comonomers (**a**) and shear stress dependencies of the viscosity (**b**), the first normal stress difference (**b**), and the relative elasticity coefficient (**c**) for PBAT melts at 190 °C. The legends indicate the comonomers’ number (adapted from [176]).

The synthesis of polymers with rare long-chain branching is of interest for enhancing the elasticity of their melts and suppressing Rayleigh instability during the formation of thin fibers and films [174,175]. In the case of the biodegradable analog of polyolefins, poly(butylene adipate terephthalate) (PBAT), polyfunctional comonomers (Figure 39a, 0.2 mol%) can provoke chain branching, resulting in polymers with comparable molecular weight characteristics (m/n = 1.1–1.2, *M*_w_ = 81–111 kDa, *Đ* = 2.0–2.4) [176]. Studies of melts show that branching comonomers increase their viscosities compared to linear PBAT (Figure 39b). This fact is neutral since altering the polymer’s molecular weight adjusts its viscosity more explicitly (*η*~*M*^3.4^). Elasticity is essential and manifests at high shear rates, accompanying the appearance of shear-thinning behavior via the emergence of normal stresses. The first difference of normal stresses (*N*_1_ = *σ*_xx_ − *σ*_yy_, where *σ*_xx_ and *σ*_yy_ are the diagonal stress tensor components along the X and Y axes and the component *σ*_xy_ is the shear stress) is also higher for branched polymers at the same shear stresses. However, it is unclear which comonomer works more effectively as a melt elasticity enhancer and whether the observed dissimilarity of samples is an effect of differences in their molecular weight and polydispersity.

The measure of melt elasticity is the coefficient of the first difference of normal stresses *ψ* = *N*_1_/$\dot{\gamma }$^2^, related to the molecular weight as *ψ*~*M*^2·3.4^ [57]. Since *η*~*M*^3.4^, the ratio *ψ*/*η*^2^ characterizes the polymer’s ability to exhibit higher elasticity at equal viscosity and is independent of its molecular weight [176]. The difference in *ψ*/*η*^2^ as the relative elasticity coefficient of PBAT melts (Figure 39c) proves the different architecture of their chains and indicates the highest branching provided by trimethylolethane.

### 6.2. Reflection of Long-Chain Polymer Branching in Linear Viscoelasticity

**Figure 40:**
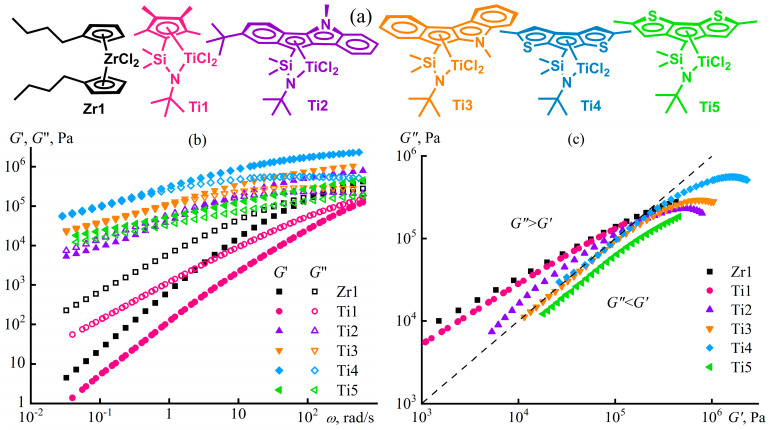
Structural formulas of pre-catalysts (**a**) and dependencies of storage and loss moduli at 150 °C on the angular frequency (**b**) and each other (**c**) for polyethylenes synthesized at 80 °C with the involvement of the specified pre-catalysts (adapted from [179]).

Measurement of normal stresses is not always possible, for example, at high melt viscosity, nevertheless leaving the analysis of linear viscoelasticity possible. Laun’s rule allows for computing the first difference of normal stresses from the data on linear storage and loss moduli: *ψ* = 2(*G*′/*ω*^2^)(1 + (*G*′/*G*″)^2^)*^a^*, where shear rate and angular frequency are numerical equal in *ψ*($\dot{\gamma }$), *G*′(*ω*), and *G*″(*ω*), while the power-law index *a* varies from 0.5 to 0.7, growing with an increase in the polydispersity of macromolecules [177,178]. Then, the ratio *ψ*/*η*^2^ can allow for assessing the branching of macromolecular chains (see Figure 39c), at least when they are comparably polydisperse for the same index *a*. However, research on the effectiveness of new pre-catalysts (Ti2–Ti5, Figure 40a) for the heterogeneous copolymerization of ethylene with 1-hexene and the production of long-chain branched polyethylene (PE) with improved melt elasticity leads to polymers having very different molecular weights and polydispersity indexes (*M*_w_ = 135–959 kDa, *Ð* = 2.3–4.1, 0.1–0.3 mol% short *n*-butyl branching), complicating the task of their comparison [179]. Dependencies *G*′(*ω*) and *G*″(*ω*) differ significantly for different PE (Figure 40b): from the typical Maxwell behavior of reference samples having linear (Zr1) or weakly branched chains (Ti1) to the low-frequency superposition of terminal zone and rubbery plateau for polyethylenes of unknown architecture (Ti2–Ti5), which is due to their high polydispersity or branching. The same data presented as Cole–Cole plots (*G*″ versus *G*′ [180], Figure 40b) are independent of molecular weight (i.e., relaxation time), frequency, and temperature, as *G*″ *=* (*G*′(*G*_0_ − *G*′))^0.5^ according to the Maxwell model, where *G*_0_ depends on the entanglement density. These plots allow for ranking the polymers by their elasticity (*G*′) at equal viscosity (*G*″) and prove the difference in chain architecture rather than polydispersity, as the dependencies *G*″(*G*′) would be identical and overlapping otherwise.

**Figure 41:**
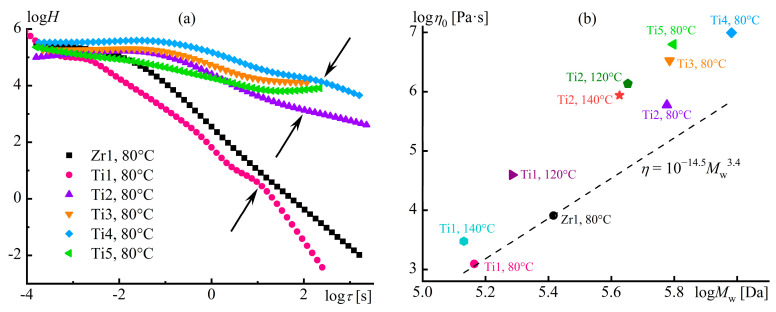
Continuous relaxation spectra for PE melts at 150 °C (**a**) and their highest Newtonian viscosity versus GPC-measured molecular weight (**b**). The pre-catalysts and synthesis temperatures are indicated in the legend and near the experimental points. The arrows show long-time shoulders associated with long-chain branching. The dashed line reflects the calculated viscosity for linear PE (adapted from [179]).

The dependencies of storage and loss moduli on angular frequency allow for computing the continuous relaxation spectra [181] of polyethylenes (Figure 41a) to refine the characteristics of their branching [179]. In logarithmic coordinates, the spectral intensity (*H*) linearly decreases at long relaxation times (*τ*) for linear PE. In contrast, the spectra of other samples have long-time shoulders (indicated by arrows in Figure 41a), more (Ti1, Ti3–Ti5) or less (Ti2) expressed, indicating a new relaxation mechanism associated with long-chain branching: either uniform and evenly distributed (Ti1, T3–Ti5) or chaotic (Ti2). Provided there are known molecular weights and close dispersities, the highest Newtonian viscosity of melts (*η*_0_~*M*^3.4^) also serves as a measure for the branching degree. The growth of chain branching raises the glass transition temperature *T*_g_ of a polymer [182], which, in turn, increases its viscosity as a glass-forming liquid due to the reduction in the difference between the glass transition and test temperatures [179]. Measurements of the viscosity of polyethylenes synthesized at different temperatures and catalysts (Figure 41b) allow for evaluating their branching as the distance on the ordinate axis in logarithmic scales between the measured viscosity and the calculated theoretical curve for linear PE. The highest deviation in the measured viscosity and the highest elasticity (Figure 40c) indicate the pre-catalyst Ti5 to be the most branch-forming for polyethylene chains.

### 6.3. Cross-Linked Macromolecular Particles and the Rheology of Their Dispersions

**Figure 42:**
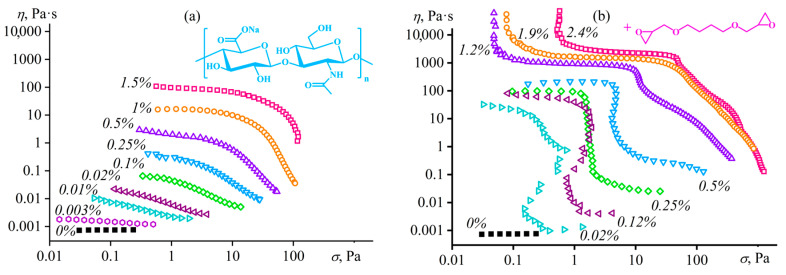
Viscosity versus shear stress for aqueous solutions of sodium hyaluronate at 25 °C (**a**) and its gels (**b**) obtained using BDDE (BDDE/SH = 1/90 wt/wt; 5 mol% disaccharide units contain cross-links) (adapted from [184,185]).

**Figure 43:**
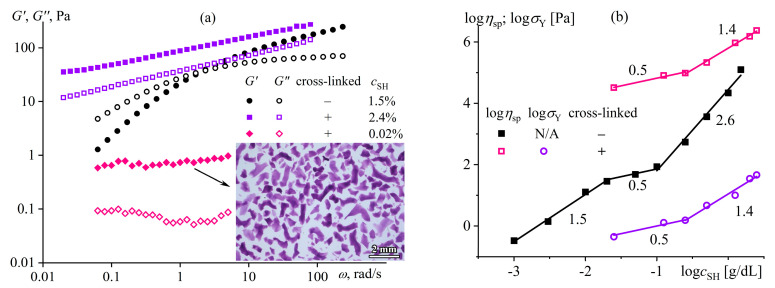
Storage and loss moduli versus angular frequency for the solution of sodium hyaluronate and its two gels (**a**) and the concentration dependencies of their specific viscosity ($\dot{\gamma }$ = 0.001 s^−1^) and yield stress (**b**) (adapted from [184,185]). Captions near the concentration dependencies indicate their slopes. The inset demonstrates an example of sodium hyaluronate’s cross-linked microgels stained with toluidine blue (adapted from [186]).

In the limit, branching leads to the cross-linked network structure of polymers with their non-flowability and insolubility. However, if cross-links do not form a single spatial network but localize inside numerous macromolecular particles, the resulting microgels can swell significantly in liquids, forming a visually homogeneous system. An example is sodium hyaluronate (SH) hydrogels cross-linked in an aqueous solution with 1,4-butanediol diglycidyl ether (BDDE), dried, ground into powder, and rehydrated for use in cosmetology and medicine as injectable implants [183]. Microbial synthesis of SH by *Streptococcus* bacteria leads to its high molecular weight (1.6 MDa) and pronounced non-Newtonian behavior in water at *c*_SH_ ≥ 0.01% (Figure 42a) [184]. The viscosity of concentrated solutions is constant at low shear stresses and decreases at high ones due to the stretching of macromolecules and a decrease in entanglement density. Concentrated hydrogels with comparable SH mass fractions (1.2–2.4%) behave similarly to the solutions at first glance [185], but have a low yield stress *σ*_Y_ = 0.1–1 Pa (Figure 42b), possibly because of the inability of gel micro-particles to move relative to each other without exerting effort to straighten their jagged shapes (see the inset in Figure 43a). Dilution of the hydrogels to 0.5–0.02% causes the disappearance of this yield stress, but another higher yield stress becomes apparent, probably associated with the destruction of the network of interparticle contacts. In more concentrated gels, this higher yield stress is inconspicuous, and their viscosity decreases at high shear stresses primarily due to the stretching of macromolecular chains.

The concentrated hydrogel demonstrates solid-like behavior (*G*′ > *G*″, Figure 43a), although its storage modulus relatively strongly depends on the angular frequency (*G*′~*ω*^0.26^), similar to CAB gels and in contrast to more stiff gels (*G*′~*ω*^0.06–0.16^). This frequency dependence may result from the polydispersity of macromolecules; uneven cross-linking; or the superposition of different networks: covalent cross-links, physical entanglements of macromolecules, and interparticle contacts. At high frequencies, the storage and loss moduli of the gel tend towards values characteristic of a non-cross-linked SH solution of comparable concentration, i.e., the moduli depend on macromolecular entanglements. In contrast, the gel’s behavior at low frequencies is determined by covalent cross-links, as its elasticity far exceeds that of the solution. Dilution of the gel weakens the dependence of the storage modulus on the angular frequency (G′~*ω*^0.08^), leaving only the action of the interparticle contacts active and making the SH hydrogel similar to other particle-based gels, such as nanocellulose ones (*G*′~*ω*^0.04–0.08^). However, further dilution disrupts the macrogel network, causing the settling of microgel particles. The maintenance of yield stress and stiffness after dilution is critical for gels as implants whose purpose is to fill the intercellular space, retain their shape, and not leak.

Concentration dependencies of the SH gel’s low-shear viscosity and high-shear yield stress (i.e., the interparticle network strength) similarly linearize in log–log coordinates, dividing the gels into semi-dilute and concentrated ones (Figure 43b). Moreover, these concentration regions correspond to those for the non-cross-linked SH solutions [184,185]. For dilute and semi-dilute SH solutions, *η*_sp_ is proportional to *c*^1.5^ and *c*^0.5^, respectively, as for usual polyelectrolyte solutions without entanglements. The gel does not exist in the dilute regime because of the absence of a percolation network from microgel particles and their sedimentation, i.e., for lack of yield stress. Cross-linking of hyaluronate macromolecules into microgels hundredfold elevates the viscosity of their dispersion because of the increase in molecular weight. However, the viscosity dependence remains the same, *η*_sp_~*c*^0.5^, which results from the decrease in the effective size of charged macromolecules with an increase in their content and, consequently, in the ionic strength of the solution. A further increase in concentration intensifies the viscosity rise because of the appearance of macromolecular entanglements, which play a more significant role in determining the rheology of the solution rather than the gel: *η*_sp_~*c*^2.6^ and *c*^1.4^, respectively.

### 6.4. Highly Branched Macromolecular Particles and Their Blends with Linear Chains

**Figure 44:**
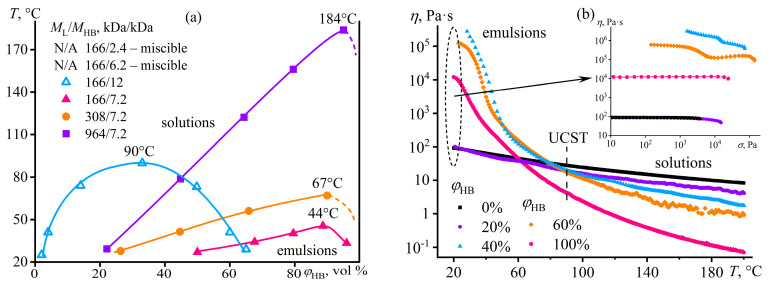
Phase diagrams for linear/highly-branched siloxane blends ((**a**), UCST is indicated near the curves) and their viscosity as a function of temperature at *M*_L_/*M*_HB_ = 166/12 kDa/kDa ((**b**), the inset shows the viscosity versus shear stress at 20 °C). The legends indicate molecular weight ratios (**a**) and volume fraction of highly branched macromolecules (**b**) (adapted from [187,189]).

The transition from microgel macromolecular particles to highly branched ones gives them fluidity at *T* > *T*_g_ and solubility, including into linear polymers of similar monomer structure, e.g., for their modification [187,188,189]. However, the energy of dispersive interactions of branched chains is lower than that of linear ones, which can lead to their immiscibility with an increase in their molecular weights and the resulting decrease in entropy’s contribution to solubility. An example is linear (L) polydimethylsiloxanes (PDMS) and their highly branched (HB) analogs—MT-resins (M/T = [Me_3_SiO_0.5_]/[MeSiO_1.5_] = 1/3.1, 75% degree of branching). Combinations of linear and highly branched siloxanes of different molecular weights can lead to both miscible and immiscible blends (Figure 44a). In this case, the increase in the molecular weight of one of the components elevates the upper critical solution temperature (UCST) and shifts the bimodal maximum along the concentration axis towards this component’s 100% content [187,189], just as for ordinary polymer blends [190,191]. These facts result in two intriguing features of immiscible blends. At the relatively low molecular weight of highly branched macromolecules (7.2 kDa), they almost do not dissolve linear macromolecules, but dissolve within them and form a dispersed phase when in excess [189]. However, at a higher molecular weight (12 kDa), highly branched macromolecules almost do not dissolve in linear ones, but dissolve them and can serve as a dispersion medium [187]. Moreover, highly branched macromolecules have higher glass transition temperatures (−53 °C and −7 °C at *M*_w_ = 7.2 kDa and 12 kDa, respectively, versus −184 °C for linear PDMS, see below), leading to a much more intense dependence of their viscosity on temperature, such that *η*_HB_ >> *η*_L_ at low temperatures and, conversely, *η*_HB_ << *η*_L_ at high temperatures (Figure 44b). These features, consisting of temperature-switchable mutual solubility, phase state, and ratio of viscosities, determine the rheological behavior of linear/highly-branched macromolecular blends.

The viscosity of miscible blends changes monotonically with a variation in the ratio of linear and highly-branched macromolecules (the right side of Figure 44b) according to the equation $\eta ={\left(\varphi_{1}{\eta_{1}}^{n}+\varphi_{2}{\eta_{2}}^{n}\right)}^{1/n}$. The exponent $n={\left(\varphi_{1}{n_{1}}^{-1}+\varphi_{2}{n_{2}}^{-1}\right)}^{-1}$ depends on the blend composition with limiting values of *n*_1_ = 0.5 and *n*_2_ = 1/3.4 for blends enriched and depleted in linear chains, respectively [187], i.e., like for a swelled polymer (*η*~*φ*^2^ [192]) and its concentrated solution (*η*~*φ*^3.4^ [193]), indicating the presence of entanglements between macromolecules of both types. The cooling of blends turns them into emulsions, which are dilute (*φ*_HB_ = 20%) or concentrated (40–60%) in the case of a relatively high molecular weight of highly branched polymer (12 kDa, i.e., insoluble in linear PDMS). The dilute emulsions are droplets of highly branched siloxane emulsified in its saturated solution in the continuous medium of linear macromolecules [187]. In contrast, the concentrated emulsions are droplets of linear PDMS saturated with highly branched macromolecules and emulsified in their medium. Remarkably, the concentrated emulsions have pronounced non-Newtonian behavior and viscosity that is in orders of magnitude higher than those of the initial polymers (inset to Figure 44b).

**Figure 45:**
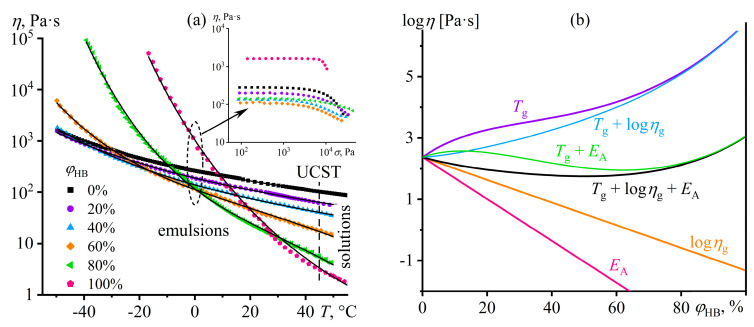
Dependencies of the viscosity of linear/highly-branched siloxane blends on the temperature at *M*_L_/*M*_HB_ = 166/7.2 kDa/kDa ((**a**); the inset shows the viscosity versus shear stress at 0 °C) and the calculations of the blend viscosity at the linear variations of the parameters indicated near the curves, the transition from linear macromolecules to highly branched ones, and 0 °C (**b**) (adapted from [189]).

The decrease in the molecular weight of highly branched macromolecules (7.2 kDa, do not dissolve linear PDMS) reverses the situation: the viscosity of emulsions is lower than that of the initial polymers, albeit within a limited temperature range only (Figure 45a). These emulsions consist of droplets of the highly branched resin in a continuous phase of its saturated solution in the linear PDMS. Upon an increase in *φ*_HB_ and a decrease in temperature, the emulsions transit from the dilute state to the concentrated one because of the reductions in both miscibility and, consequently, the proportion of the dispersion medium. An anomalously low viscosity is characteristic of dilute emulsions whose viscosity depends primarily on the viscosity of the continuous phase (neglecting the viscoelasticity of the interfacial boundary, not manifesting itself visibly). The approximation of the temperature dependencies of the viscosities of the linear and highly-branched macromolecules shows that they adhere to only one of the equations—Arrhenius–Frenkel–Andrade [194,195,196] or Williams–Landel–Ferry [197], respectively, which can be due to the different dominant factors determining their viscosity, i.e., macromolecular entanglements or glass transition [189]. A mean-square averaging over temperature can combine both equations to describe the blends’ viscosities: $\log\frac{\eta }{\eta_{g}}=-\left(\frac{c_{1\;}(T-T_{g})\;}{c_{2}+(T-T_{g})\;}\cdot \frac{T_{0}^{2}}{{\left(T-T_{g}\right)}^{2}}+\frac{E_{A}(T-T_{g})\;}{2.303RTT_{g}\;}\cdot \frac{{\left(T-T_{g}\right)}^{2}}{T_{0}^{2}}\right)/\left(\frac{T_{0}^{2}}{{\left(T-T_{g}\right)}^{2}}+\frac{{\left(T-T_{g}\right)}^{2}}{T_{0}^{2}}\right)$, where *η*_g_ represents the viscosity at the glass transition point, *R* is the universal gas constant, *E*_A_ is the activation energy of viscous flow, and *T*_0_ is the temperature of the relative transition from the dominance of glass forming ((*T* − *T*_g_) < *T*_0_) to the dominant role of macromolecular entanglements (*T* >> *T*_g_). This equation describes the viscosity of blends of any composition (black lines in Figure 45a), allowing for their characteristics (*T*_g_, log*η*_g_, *E*_A_) to be determined and rheological behavior to be modeled when varying the component ratios (Figure 45b). The transition from linear macromolecules (−184 °C, 15.6 [Pa·s], 30.6 kJ/mol) to highly branched ones (−53.2 °C, 11.9 [Pa·s], 97.9 kJ/mol) with the corresponding change in the above-indicated characteristics passes through a viscosity minimum only when *T*_g_ and *E*_A_ of their resulting blend change simultaneously and in the same direction. Furthermore, the accompanying opposite change in log*η*_g_ intensifies the effect of the abnormal viscosity decline and extends it across the broader range of the blend composition. Thus, a combination of a polymer having high *E*_A_ and *T*_g_ with a miscible polymer exhibiting lower *E*_A_ and *T*_g_ values reduces the viscosity of their blend at certain temperatures, which can intensify the processing of polymers in the plastic industry.

## 7. Structured Polymer Materials

**Figure 46:**
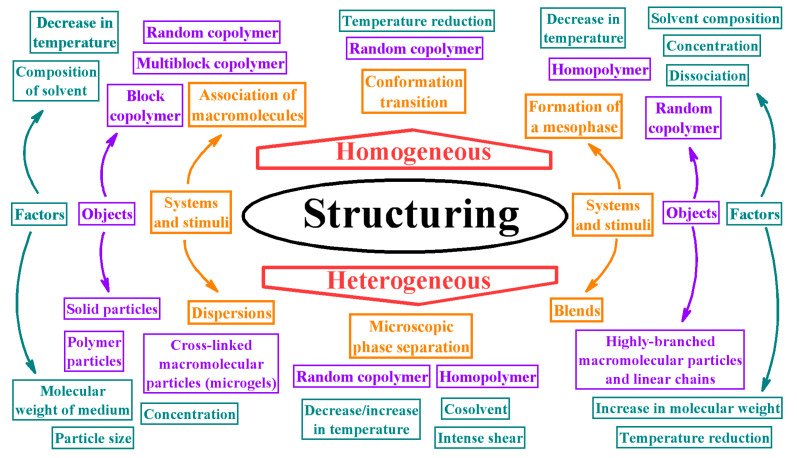
Generalized scheme of structure formation in macromolecular systems.

Summarizing the results of the rheological analysis of complex macromolecular systems, structure formation can be homogeneous and heterogeneous (Figure 46). Homogeneous structuring may result from the association of copolymer macromolecules, conformational transitions, or a combination of these causes, leading to mesophase formation. Typically, a decrease in temperature favors structure formation, increasing the stiffness (storage modulus) and strength (yield stress) of the resulting system. Moreover, the dissociation of macromolecules, their concentration, and the solvent composition are additional factors influencing the temperature of structure formation and its degree. Heterogeneous structuring may occur because of the microscopic phase separation of a polymer solution upon a temperature change or the addition of a nominal cosolvent, and the intense shear may either promote or hinder phase separation. Structure formation can also happen in dispersions of solid particles in polymeric media, enhancing with a rise in the volume fraction of particles and a reduction in their size and weakening as the molecular weight and concentration of macromolecules increase. Dispersions of swelled polymer particles and cross-linked microgel particles also form structures at moderately high concentrations, gaining yield stress behavior and sedimentation stability. Furthermore, structuring is possible in polymer blends, even in mixtures of linear and highly-branched macromolecules with similar chemical structures at sufficiently high molecular weights and low temperatures. Since structure formation is typical for most complex macromolecular systems and occurs under certain conditions, structural rheology as a method for characterizing structures can facilitate the development of functional polymer materials, which usually involves polymer melts, solutions, and gels as bases for creating polymer composites, adhesives, fibers, membranes, greases, and other functional materials.

### 7.1. Composite Pressure-Sensitive Adhesives

**Figure 47:**
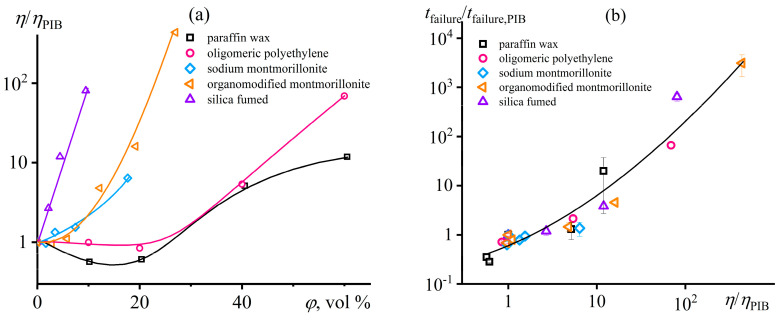
The viscosity of the poly(iso)butylene-based pressure-sensitive adhesives as a function of the volume fraction of dispersed particles (**a**) and the increase in time to failure of adhesive bonds of these adhesives with steel as a function of their viscosity at 25 °C (**b**) (adapted from [157,202,203,204]).

Thermoplastics, elastomers, and thermosets can serve as matrices for composites and adhesives when their rheological characteristics in the flow or high-elastic state indirectly characterize the size of dispersed particles, their structuring, and the material’s behavior under various action rates. For instance, the issue of pressure-sensitive adhesives (PSAs [198,199]) having no cross-links lies in creep behavior or cold flow [200,201], the overcoming of which can consist of obtaining composite PSAs [157,202,203,204]. Dispersed particles increase the viscosity of the PIB-based PSA because of the adsorption of polymer chains on their surfaces and the growth of their effective molecular weight (see Figure 34 and its discussion). This effect is higher when the surface area of the particles is greater, i.e., their volume fraction is larger and their dispersion in the polymer matrix is better, increasing the proportion of the interphase layer. A comparison of increments in the relative viscosity of the PSA filled with particles of different natures (Figure 47a) reveals the most pronounced thickening effect of silica nanoparticles (20 nm). In contrast, paraffin wax or oligomeric PE can even decrease viscosity at low mass fractions because of their modest solubility within the PIB medium. Nonetheless, regardless of the nature or size of the particles, there is a clear correlation between the time to failure of the adhesive bond under shear and the viscosity of the composite adhesive (Figure 47b), allowing for improvement of this operational characteristic by four decimal orders of magnitude with both micro- and nanoparticles, but at a lower consumption of the latter.

### 7.2. Asphaltenes as Tackifiers for Hot-Melt Adhesives

**Figure 48:**
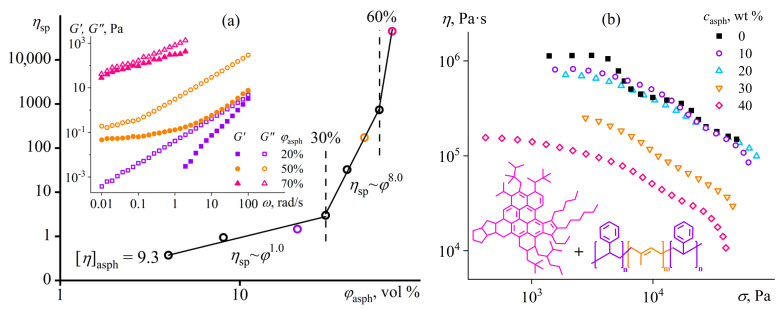
The specific viscosity for asphaltenes (*M*_w_ = 828 Da) dissolved in diisooctyl sebacate at 25 °C ((**a**); the inset shows the frequency dependencies of the storage and loss moduli of the solutions) and the viscosity at 120 °C for SIS melts (44% styrene units, 82 kDa) containing asphaltenes (**b**). The legends indicate asphaltene mass fractions (adapted from [211,219]).

Usual components of polymer adhesives are tackifiers—miscible oligomeric compounds providing polymers with liquid-like behavior at elevated temperatures (hot melts [205]) or prolonged times (PSAs [198,199]), e.g., hydrocarbon resins [206,207]. An alternative to the latter can be highly branched macromolecular particles, for instance, asphaltenes—the highest-molecular-weight components of heavy crude oils and bitumens [208,209]. Asphaltenes are soluble in weakly polar organic media (when the solubility parameter *δ* > 16.5 MPa^0.5^ [210]), for example, diisooctyl sebacate (DOS, 17.5 MPa^0.5^), forming dilute, semi-dilute, and concentrated solutions [211], each with their characteristic dependencies of the specific viscosity *η*_sp_(*φ*) (Figure 48a). The dimensionless intrinsic viscosity [*η*] of asphaltenes is 9.3 (equal to 0.078 dL/g [211]), differing from 2.5 for spherical particles in the Einstein equation *η*_sp_ = [*η*]*φ* [212,213] and indicating the self-assembly of asphaltenes into extended nanoaggregates even in dilute solutions through π–π interactions of their polyaromatic cores [208,209]. Asphaltene solutions are viscoelastic (the inset in Figure 48a) [211], demonstrating the Maxwellian behavior of dilute solutions due to the proximity of the test and glass transition temperatures (weighted harmonic mean according to the Fox equation [214], *T*_g,asph_ = 63 °C, *T*_g,DOS_ = −105 °C) and the structuring of semi-dilute (*G*′ ≈ *const* at *ω* → 0) and concentrated solutions through interasphaltene interactions. Viscoelasticity and the three concentration zones of the solutions make asphaltenes phenomenologically akin to linear macromolecules. However, the reasons for these rheological phenomena are nanoaggregation and glass transition of asphaltenes rather than macromolecular entanglements and high elasticity.

**Figure 49:**
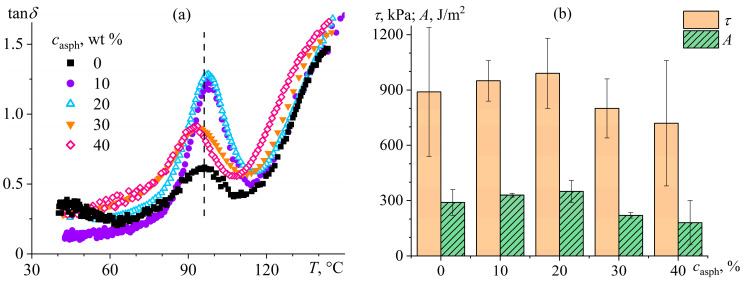
The loss tangent versus temperature for the asphaltene-containing SIS at *ω* = 6.28 rad/s (**a**) and the adhesion strength and apparent work of its adhesive bonds with a steel surface upon pull-off at 25 °C (**b**) (adapted from [219]). The dashed line represents the glass-transition temperature for pure SIS.

Asphaltenes (*δ* = 19–22 MPa^0.5^ [215]) are partially soluble even in polymers [216,217,218], for example, in the SIS (16.7 and 22.5 MPa^0.5^ for isoprene and styrene blocks, respectively [140]), used as a base for hot melts and PSAs [219,220]. The SIS melt separates into microphases (see Figure 7), and its viscosity, depending primarily on the rigid styrene blocks, remains almost unchanged after adding 10–20% asphaltenes (Figure 48b), indicating their dissolution within the isoprene blocks [219]. Viscosity reduction happens at 30–40% asphaltenes that plasticize the styrene blocks, confirmed by the decrease in their *T*_g_ (indicated by the vertical line in Figure 49a) by 2–4 °C. In contrast, lower asphaltene content raises *T*_g_ by 2 °C, enhancing block segregation and elevating tan*δ* at the maximum (Figure 49a) because of the increased volume fraction of isoprene blocks containing dissolved asphaltenes. These transformations result in *G*″/*G*′ > 1, which provides liquid-like behavior to the hot-melt adhesive in a specific temperature range and, consequently, its better tack, reflected in the increased strength (*τ*) and apparent work (*A*) of adhesion (Figure 49b). Higher asphaltene content penetrates the styrene microphase, increasing its volume fraction, reducing tan*δ*, and worsening the tack and adhesive performance.

### 7.3. Cellulose Membranes for Nanofiltration

In the case of polymer solutions, their possible structuring is influential when producing membranes and fibers [221,222]. Phase separation of polymer solution allows for obtaining porous membranes for filtering liquid media and results from changes in temperature, evaporation of solvent, or exposure to non-solvent in the form of vapor or liquid [17]. A common task of rheology is to choose the polymer concentration in the solution to ensure macromolecular entanglements and a specific viscosity according to technological regulations. However, rheology also allows for determining the maximum possible non-solvent content in the shaping solution and the features of phase separation (Figure 14, Figure 17 and Figure 18), thus enabling the reduction in its duration upon contact with the non-solvent and, consequently, decreasing the resulting pore size, which is essential for obtaining nanofiltration membranes [130].

**Figure 50:**
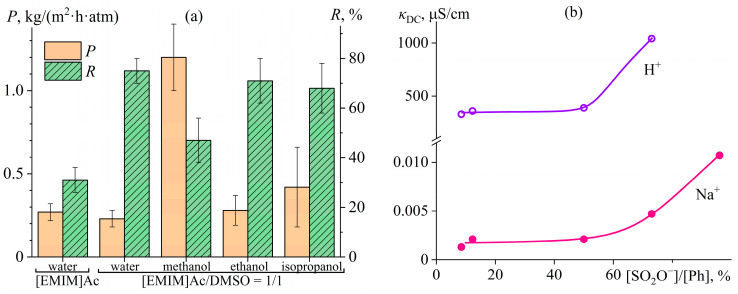
Permeability of DMF and retention coefficient for a model pollutant (Remazol Brilliant Blue R, 626 g/mol) when using cellulose nanofiltration membranes ((**a**), the precipitant and solvent are on the abscissa scale) and the electrical conductivity of polyoxadiazole films versus their sulfonation degree and the type of cation (**b**) (adapted from [130,137]).

For example, the addition of 50% DMSO to [EMIM]Ac reduces the viscosity of the 14% cellulose solution (Figure 17a) to produce asymmetric composite membranes, facilitating the impregnation of the nonwoven substrate from poly(ethylene terephthalate) and accelerating the subsequent diffusion of precipitant molecules—water. In addition, the quality of the ionic solvent worsens with a decrease in the amount of precipitant that initiates phase separation. As a result, the phase separation accelerates, leading to the more rapid formation of a dense selective surface layer of the membrane, reducing the pore size within it and doubling the retention coefficient (*R*, Figure 50a) for the large molecules of a model pollutant—anthraquinone-based dye dissolved in DMF. Meanwhile, the permeability of DMF (*P*) remains unchanged, indicating the constancy of the porosity of the membrane’s supporting layer, i.e., the growth of retention due to the increased density of the selective layer. The precipitation in water induces phase separation through the stage of gel formation (see Figure 18), slowing down the structural reorganization of the disintegrating solution. In contrast, the gradual addition of an aliphatic alcohol to the cellulose solution reduces its viscosity until it causes the precipitation of the polymer. Prevention of the gel formation during membrane formation by precipitation with methanol accelerates the growth of pores, increasing the permeability fivefold but reducing the retention coefficient (Figure 50a). At the same time, an increase in the hydrophobicity of a precipitant using ethanol or isopropanol enhances the shrinkage of the forming membrane, reducing the pore size and thereby elevating the retention coefficient, but at the expense of permeability [130].

### 7.4. Ion- and Proton-Exchange Polyoxadiazole Membranes

The choice of solvent and precipitant, taking their influence on the viscosity and phase state of the solution into account, allows for modifying the transport characteristics of porous membranes. In the case of non-porous ion-exchange membranes made from sulfonated polyoxadiazoles, a rheological analysis (Figure 20, Figure 21 and Figure 22) enables the selection of the solvent, temperature, and polymer concentration for producing membranes by casting the polymer solution or impregnating a nonwoven substrate by it with subsequent drying [137], which is impossible using solutions in sulfuric acid. The electrical conductivity of membrane films from the initial sodium polyoxadiazoles increases with the content of sulfonate groups (Figure 50b), reaching 1.07 × 10^−8^ S/cm at direct current for the most sulfonated polymer (P0, Figure 19). The ionic conductivity of polymers with comparable thermal stability is usually much lower, e.g., 10^−13^ S/cm for sulfonated poly(*p*-phenylene) (SO_2_OLi/Ph = 1/2) [223]. The treatment of polyoxadiazoles with a 0.1 M solution of HCl converts them to the acidic form, allowing composite proton-exchange membranes to be obtained with a five-order-of-magnitude rise in conductivity. The trend of the growth in the conductivity with the increasing degree of sulfonation persists up to 1.04 × 10^−3^ S/cm (direct current, 25 °C, 20% relative humidity), i.e., to a value comparable to the conductivity of sulfonated polytetrafluoroethylenes of the Nafion family [224]. However, sulfonated polyoxadiazoles are more thermally stable since their thermal decomposition starts at 460–475 °C versus 280–400 °C for sulfonated PTFEs [137].

### 7.5. Nanocellulose Oleogels as Lubricating Greases

Polymer gels are inherently structured, and their structure determines their elasticity (*G*′), strength (*σ*_Y_), and, consequently, their range of applications, which vary most widely in the case of lubricating materials [225,226]. Different friction rates and regimes require lubricants with appropriate consistency and viscosity. The most heavy-loaded boundary friction regime demands lubricating greases (i.e., having a yield stress), whose task is to reduce the wear of the contacting bodies besides the decrease in their friction. Thickener particles hinder wear, also determining the rheological properties of greases and, consequently, their applicability. The wear rate of a body is proportional to the hardness of the counterbody, making soft particles of cellulose-based oleogels promising thickeners for lubricating greases. Such an oleogel can be of two types: dispersed nanocellulose particles (e.g., MFC or RC [168,169], Figure 37) or a continuous high-elastic phase of a swollen cellulose derivative (CAB, Figure 25) formed because of the microscopic phase separation of its solution, which may also include solid particles (Figure 33) [146].

**Figure 51:**
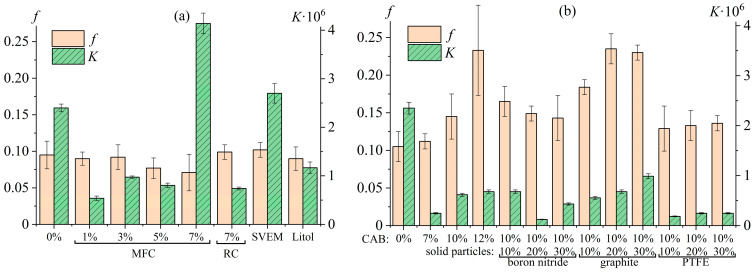
Friction and wear coefficients when testing lubricating greases based on TEC and nanocellulose (**a**) or ATBC and cellulose acetate butyrate (**b**) at 25 °C and a contact pressure of 1910 MPa. The cellulose-based thickener mass fraction and the presence of solid particles are indicated on the abscissa scale. Data for Litol and SVEM greases are provided for comparison (adapted from [146,168,169,227]).

Tribological studies of a steel ball/disc pair sliding in boundary conditions and lubricated with pure oils (TEC, ATBC) or cellulose-based gels show a significant reduction in the wear (*K*, Figure 51a) without changing the friction (*f*) upon the addition of as low as 1% MFC to TEC due to the filling of surface roughness by nanocellulose [168]. An increase in the MFC content up to 7% slightly reduces the friction thanks to the grinding of steel surfaces, but raises the wear because of the abrasive action of microfibrils. The transition from MFC to RC of the same mass fraction lowers the wear because of the amorphous nature of RC, but without reducing the friction [169]. Nanocellulose greases are comparable in antifriction action to commercial greases Litol and SVEM based on lithium 12-hydroxystearate dispersion in hydrocarbon or ester medium, respectively [227], but surpass them in antiwear activity.

### 7.6. Greases Based on Cellulose Acetate Butyrate

Compared to colloid nanocellulose gels, polymer gels based on CAB reduce wear more substantially, but increase friction (Figure 51b) [146]. The friction rise may result from the lower tendency of high-viscosity CAB gels to wall slip compared to nanocellulose dispersions (see Figure 37d), while the more efficient reduction in wear is due to the lower hardness of the swollen CAB phase. The addition of solid particles justified the latter conclusion. Boron nitride particles unite with the CAB gel network (Figure 33e), reducing the wear. Conversely, graphite particles disrupt the polymer gel network and create their own (Figure 33f), increasing wear and friction. PTFE particles are inert and act as antifriction and antiwear fillers for the gel (Figure 33g), ultimately improving both tribological characteristics. In this way, fundamental studies of the rheological behavior of structured polymer melts (e.g., prospective adhesives), solutions (as membrane precursors), and gels (potential greases) allow for their structure to be assessed in order to create and improve specific materials and address practical challenges.

## 8. Conclusions

The fundamental relationships between the rheological properties of complex macromolecular systems and their micro- and nano-heterogeneous structures are the basis of structural rheology. In turn, structural rheology, allowing us to infer the structure of a material from its rheological properties, is a valuable tool for creating and studying new functional polymer materials with enhanced characteristics, including greases, membranes, and adhesives, revealing the following conclusions:

- Growth of chain branching raises the viscosity and, even more so, the elasticity of the polymer melt and ultimately leads to limited miscibility with linear chains of similar monomer structure because of the increases in glass transition temperature, entanglement density, and enthalpy contribution into miscibility.
- Highly branched macromolecular particles, unlike ordinary colloid particles, may be miscible with low- and high-molecular-weight media but, unlike chain macromolecules, do not exhibit a rubbery-like state, and their viscoelasticity is due to glass transition rather than a physical network of entanglements.
- Linear chains are much more willing to dissolve small isomonomer molecules of highly branched architecture within themselves than dissolve in them. As a result, linear chains form a dispersion medium even with an excess of highly branched macromolecules, leading to abnormally low viscosity of the arising emulsion at specific temperatures.
- Large highly branched macromolecules more easily dissolve isomonomer linear chains than dissolve within them. At a moderate excess of the linear chains, this specific feature can lead to the formation of extremely high-viscosity concentrated emulsions of the linear polymer in a glass-forming highly branched dispersion medium.
- A mixing of miscible polymers, one of which has high flow activation energy and glass transition temperature while the other has low, is a way to reduce the viscosity of their melts for the purpose of intensifying their processing.
- Specific interchain and supramolecular interactions in polymer solutions and melts lead to structure formation, i.e., to yield stress and solid-like behavior—the gel formation. However, the dominance of macromolecular entanglements in determining rheological properties may mask these interactions that become evident only at long observation times.
- Large-amplitude oscillatory shear may lead to phase separation of polymer solutions, which is unattainable via continuous shear due to the Weissenberg effect.
- Dispersed particles act as adsorption sites for macromolecules, sharply increasing their apparent molecular weight and competing with macromolecular entanglements for the right to determine the rheological properties: a particle coagulation network or a macromolecular entanglement network. Decreases in the particles’ volume fraction and specific surface area and an increase in the polymer’s molecular weight induce a transition from the first extreme to the other.
- The transition from micro- to nanostructures for organizing macromolecules and improving applied properties is unreasonable, as their structuring operates on a microscopic scale in contrast to the unstructured nanoscale state of their dilute solutions. However, the passage from micro- to nanoparticles as modifiers of polymer media has the sense to reduce the particle doses necessary for chain adsorption and the formation of an interphase polymer layer with enhanced properties.
- The properties of applied polymer materials (adhesives, greases, membranes, etc.) depend on the structure of their precursors—polymer melts, solutions, and gels, which can undergo structural–rheological analysis for subsequent targeted modification of the composition and characteristics of the final materials.

Further development of structural rheology of polymer materials requires investigating the anisotropy in the rheological properties of complex macromolecular systems and their behavior under uniaxial extension and the application of electric and magnetic fields, as well as the use of direct methods of structural analysis during their flow and deformation, which will provide additional information about their micro- and nanoheterogeneous structures and enrich the polymer science.

## Data Availability

The data presented in this study are available upon request from the corresponding author.
